# Adaptive Catalytic Nanointerfaces for Controlled Hydrogen Evolution: an in Situ Electrochemical Approach

**DOI:** 10.1002/advs.202505104

**Published:** 2025-05-23

**Authors:** Carlos Herreros‐Lucas, Melanie Guillén‐Soler, Lucía Vizcaíno‐Anaya, Glen Murray, Mehtap Aygün, José Manuel Vila‐Fungueiriño, María del Carmen Giménez‐López

**Affiliations:** ^1^ Centro Singular de Investigación en Química Biolóxica e Materiais Moleculares (CiQUS) Universidade de Santiago de Compostela Santiago deCompostela 15782 Spain; ^2^ School of Chemistry The University of Nottingham University Park Nottingham NG7 2RD UK

**Keywords:** confined electrocatalyst, electrochemical switching, hydrogen production, reconfigurable step‐edge

## Abstract

Precious metal nanoparticles in electrocatalytic applications tend to be single‐use, becoming unusable afterward. Here, this is demonstrated that the electrocatalytic response of these nanoparticles, when confined at the step‐edges of corrugated carbon nanofibers interiors, can be switched on again at will by simply introducing sulfur as an inorganic mediator. To achieve this, an electrochemical methodology is developed that triggers the rapid surface reconfiguration of confined, deactivated nanoparticles (PdS_x_) involving the release of sulfur to yield highly active crystalline Pd(0) nanoparticles, confined polysulfides, and sulfur‐terminated carbon step‐edges. More importantly, the electrochemical performance can be systematically switched from a highly active mode, in which polysulfides enhance the hydrogen adsorption on palladium, to a much less active mode, called the resting mode, in which sulfur (formed by the oxidation of polysulfides) passivates the active Pd(0) nanoparticle surface. This discovery introduces a new protocol to control nanoparticle performance for catalytic reactions, and more crucially, to extend their lifespan.

## Graphical abstract

The ability to switch the HER activity of precious metals at will through a reconfigurable step‐edge/nanoparticle interface, using sulfur as an inorganic mediator, is demonstrated for the first time. This methodology addresses the durability issue of highly catalytic precious metals, which are typically single‐use, thereby extending their lifespan.

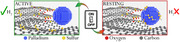

## 1 Introduction

In recent years, a great deal of interest has arisen to produce hydrogen from water electrolysis coupled with renewable sources in an effective manner.^[^ ^1^ ^]^ Large‐scale hydrogen production, as a clean and renewable energy vector, greatly depends on the system used as an electrocatalyst.^[^ ^2^ ^]^ Thus, the production of green hydrogen is mainly restricted by two problems: first, the use of expensive and scarce precious metal‐based electrocatalysts supported on carbon; and second, the lack of durability of these electrode materials under typical working conditions. During long‐term operation, electrochemical carbon support degradation, characterized by irreversible conductivity loss and reduced active area, leads to precious metal nanoparticle agglomeration and decreased electrochemical surface area.^[^ ^3^ ^]^ As a result, these electrocatalyst systems tend to be single‐use.

In an operating system, the ability of an electrocatalyst to switch its activity ON/OFF in a short timescale is very attractive, as it can further extend its use in commercial applications.^[^ ^4^ ^]^ In this context, the reconfiguration of the electrode during an electrochemical process can address the problem of durability by tuning the active sites.^[^ ^5^ ^]^ Many examples have been reported in the literature where the electrocatalyst material is activated or its surface reconfigured under working conditions, exposing the electrochemical active sites. In most cases, these involve modifications in the morphology, size, and crystal structure of the active catalyst;^[^ ^6^ ^]^ incorporation of oxygen atoms into a metal chalcogenide;^[^ ^7^ ^]^ or surface reduction/oxidation processes,^[^ ^8^ ^]^ among other strategies. However, very few examples have demonstrated an induced activation mechanism involving the reconfiguration of the catalyst support itself. The chemical nature of the selected carbon support may play an active role in electrocatalytic processes though metal‐support interactions.^[^ ^9^ ^]^ In hybridized electrocatalytic systems, Gimenez‐Lopez and co‐workers demonstrated that electrochemical activation of the catalyst can be also induced by transforming the surface of the carbon support.^[^ ^10^ ^]^ Hollow graphitized carbon nanofibers (CNF) contain corrugated interiors formed by graphene nanocone stacks leading to folded step‐edges where nanoparticles can be stabilized.^[^ ^11^ ^]^ During hydrogen evolution reaction (HER) operating conditions, these step‐edges become unfolded, enabling an increase in the HER activity.

Herein, we report for the first time the electrocatalytic activation of poorly active PdS_x_/CNF, where amorphous palladium sulfide nanoparticles are confined in CNF and assembled on their folded carbon step‐edges that can be reconfigured (unfolded) by electrochemical cycling during HER. A tailored electrochemical protocol was implemented here to achieve the in situ sulfur functionalization of step‐edge sites within the corrugated interiors of CNF, coupled with polysulfide species formation. These species exhibit dual interaction capabilities: i) coordination with Pd nanoparticles and ii) binding to reconfigured step‐edges. This dynamic interfacial chemistry enables reversible switching of the electrocatalyst between a low‐activity (resting) state and a high‐activity (active) state. The active reconfiguration mode of the electrocatalyst material presents excellent kinetics toward HER with a Tafel slope of 34 mV dec^−1^ and very low overpotential of 22 mV. Then, the material can return to an inactive mode, which is different from the mode of the initial material. This switching ability is reversible through the proper electrochemical procedures that were developed in this work. In particular, the transformations of the carbon support and the surface of the nanoparticles during the electrochemical tests are key for the reversibility of this process.

## 2 Results and Discussion

### 2.1 Preparation and Structural Characterization of PdS_x_/CNF

**Figure 1:**
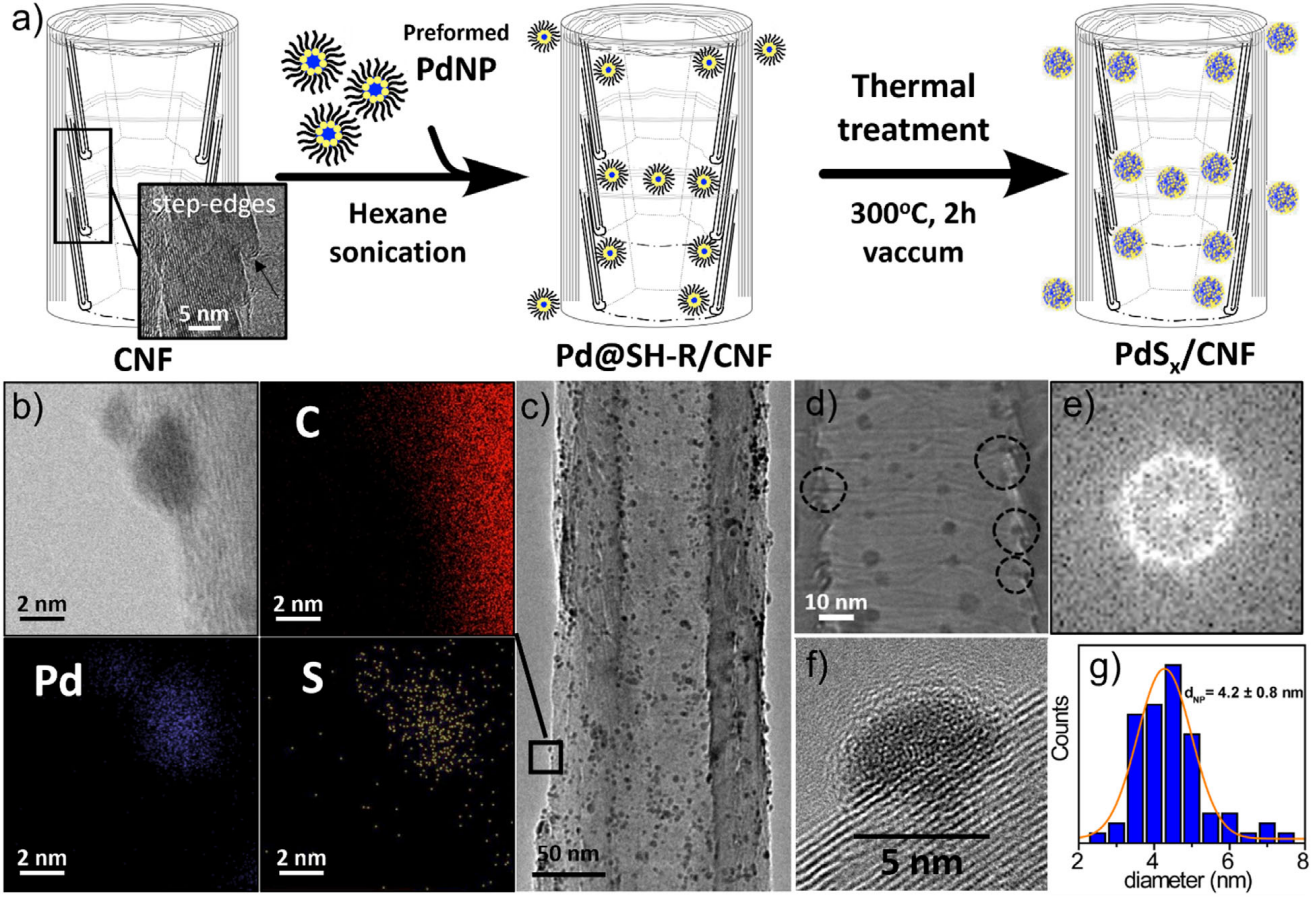
Synthesis and characterization of PdS_x_/CNF. a) Preparation scheme of PdS_x_/CNF b) STEM‐ EDS mapping images of PdS_x_ nanoparticles located outside CNF in PdS_x_/CNF in which C (red), Pd (blue), and S (yellow) atoms are presented. c) HRTEM image of the final PdS_x_/CNF material. d) HRTEM image of amorphous PdS_x_ particles located at the step‐edges (dash circle). e) FFT of a representative PdS_x_ nanoparticle. f) HRTEM image of a PdS_x_ particle outside CNF. g) Size distribution histogram and Gaussian fitting of PdS_x_ nanoparticles.

To ensure the decoration of hollow CNF with palladium sulfide nanoparticles, a three‐step experimental procedure was followed (**Figure** **1**a). First, preformed Pd nanoparticles (Pd@SH‐R) were synthesized via a modified Brust–Schiffrin protocol, which enables the formation of small and spherical thiolate‐protected nanoparticles (average size of 2.1 ± 0.7 nm) through a radical dissociative chemisorption process induced by Pd (Figure S1,S2, Supporting Information).^[^ ^12^ ^]^ By simple sonication in hexane, these preformed palladium nanoparticles were then adsorbed onto the carbon surface of CNF, which had been previously milled to reduce electrolyte transport resistance while maintaining its microscale length to prevent leaching (CNF, Figure S3, Supporting Information). The resultant material Pd@SH‐R/CNF was subsequently heated (300 °C for 2 h) in vacuum leading to the cleavage of the C‐S bond and a successive formation of Pd‐S bonds. The final material PdS_x_/CNF was carefully characterized by microscopy, spectroscopy, and thermogravimetric techniques.

Combined energy dispersive X‐ray and scanning transmission electron microscopy (STEM‐EDS) techniques were used to map sulfur and palladium in the PdS_x_/CNF material. As shown in Figure 1b and Figure S4 (Supporting Information), the presence of sulfur in the Pd nanoparticles can be confirmed. The PdS_x_ nanoparticles that were homogeneously distributed both inside and outside along the carbon nanofibers retained a small size (4.2 ± 0.8 nm) despite the treatment at 300 °C as shown by high‐resolution transmission electron microscopy (HRTEM) (Figures 1c). The size limitation of nanoparticles located inside CNF was expected based on previous studies, which demonstrated that the corrugated surfaces (i.e., the interior of CNF) can stabilize nanoparticles under harsh conditions better than smooth carbon surfaces (i.e., the exterior of pristine CNF).^[^ ^13^ ^]^

**Figure 2:**
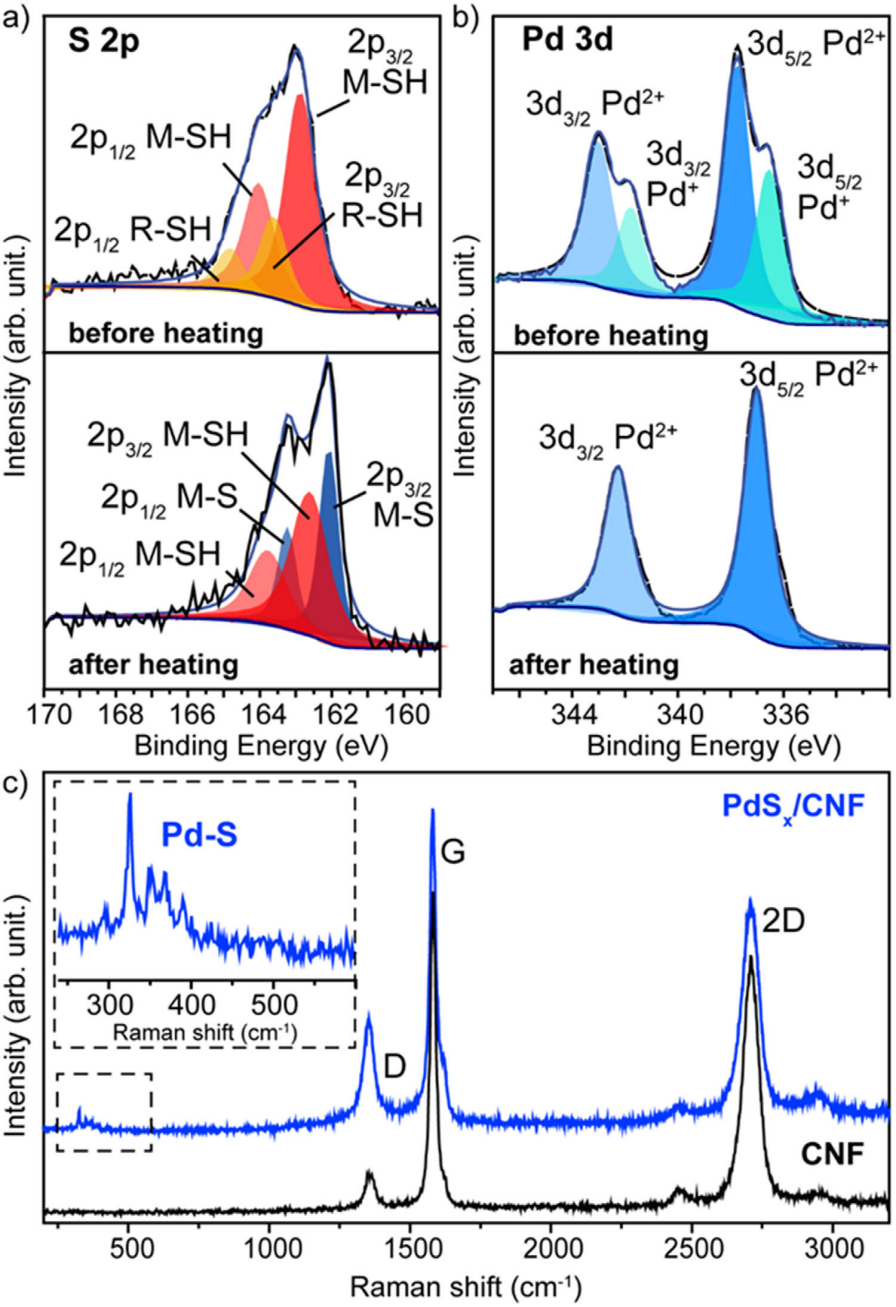
High resolution XPS spectra of a) S 2p and b) Pd 3d regions in Pd@SH‐R/CNF (before heating) and PdS_x_/CNF (after heating). Charge correction for the C 1s spectrum was set at the binding energy of 284.80 eV. In the composite before heating, the S 2p region exhibits two components: the 2p_3/2_ binding energy of 162.87 eV is assigned to the thiolate species bonded to the metal in the assembled monolayer,^[^ ^16^ ^]^ while the peak at 163.49 eV belongs to the alkylthiolate groups (R‐SH). In contrast, in the composite after heating, a new component of 2p_3/2_ appears at a binding energy of 162.06 eV which is correlated to the sulfur present in a metal sulfide phase.^[^ ^17^ ^]^ c) Raman spectrum of PdS_x_/CNF showing a D, G, and 2D band at 1350, 1550, and 2700 cm^−1^, having a I_D_/I_G_ of 0.25.

Hence, the presence of step edges inside CNF provides anchoring sites for metal nanoparticles, which increase the stability of PdS_x_ particles present in PdS_x_/CNF material (Figure 1d, dashed circles). Surprisingly, small nanoparticles were also located on the exterior of CNF after thermal treatment, which indicates that defects formed on the surface of CNF during ball milling (Figure S5, Supporting Information) can play a role in the stabilization of nanoparticles. To demonstrate that the observed size limitation is related to the roughness of the external carbon surface, Pd nanoparticles (Pd@SH‐R) combined with pristine CNF (with a smooth exterior) were thermally treated. A two‐size nanoparticle distribution was observed (small nanoparticles inside and large nanoparticles outside, Figure S6, Supporting Information), corroborating that the increase in the CNF surface roughness due to ball milling facilitates the stabilization of nanoparticles, limiting their growth. Fast Fourier Transform (FFT) obtained from HRTEM shows that the PdS_x_ nanoparticles inside and outside of the CNF have an amorphous nature, as no clear lattice fringes were observed (Figure 1e). Taking into consideration the employed preparation method,^[^ ^14^ ^]^ which involves first a fast reduction step followed by a radical dissociative chemisorption process of adsorbed thiolates promoted by the Pd after heating, this amorphous nature could be explained.^[^ ^15^ ^]^ It is worth noting that the characterization of PdS_x_/CNF was very challenging due to the very small size of nanoparticles, as discussed above. For that reason, and to confirm the palladium sulfide nature of the nanoparticles, PdS_x_/CNF was studied by X‐ray photoelectron spectroscopy (XPS) and compared with the composite before heating (Pd@SH‐R/CNF). In the XPS survey spectra of both samples the C 1s, O 1s, S 2p, and Pd 3d regions were identified (Figure S7, Supporting Information). The appearance of the metal sulfide component (S(2p_3/2_) M‐S), along with the disappearance of the R‐SH peak (S(2p_3/2_) R‐SH) in PdS_x_/CNF, indicates that after heating up the composite (Pd@SH‐R/CNF), strong Pd‐S bonds were formed (**Figure** **2**a).^[^ ^16^ ^]^ No oxidation of sulfur was observed in the S 2p XPS signals for any of the composites. On the other hand, in Pd@SH‐R/CNF two components were deconvoluted in the Pd 3d core‐level spectra at binding energies of 337.76 eV and 336.50 eV, both assigned to a non‐zero valent state of Pd that is in agreement with oxidized Pd single atom or small clusters previously reported.^[^ ^18^ ^]^

After heating, the high‐resolution Pd 3d core level spectra exhibited only one type of non‐zero valent state at a binding energy of 337.0 eV (Figure 2b) that could be assigned to the formation of a PdS phase.^[18c]^ This is consistent with the results of S 2p XPS signals. The binding energies and the relative concentrations of the sulfur and palladium species are listed in Table S1 and S2 (Supporting Information). Hence, all of the aforementioned analyses provide confirmation of the production of metal sulfide (Table S3, Supporting Information). These findings are consistent with Raman assessments carried out on PdS_x_/CNF, which display five peaks between 300 cm^−1^ and 400 cm^−1^, denoting high‐energy phonons inherent to PdS (Figure 2c).^[^ ^19^ ^]^ It is noteworthy that neither Pd‐O nor C‐C stretching bands were observed at 650 cm^−1^ and 1080 cm^−1^ in the spectrum (Figure 2c). Thermal gravimetric analysis (TGA) of PdS_x_/CNF, compared with preformed Pd nanoparticles in air, showed no weight loss at ≈125 °C (as in preformed PdNPs, Figure S1, Supporting Information), only at higher temperature (i.e., ≈290 °C) (Figure S8, Supporting Information).

In view of these results, a novel hybrid material, PdS_x_/CNF, has been prepared in this work, presenting a unique opportunity to investigate a reconfigurable interphase effect between a carbon‐based step‐edge and a sulfur‐doped nanoparticle system for the HER.

### 2.2 Electrochemical Activation of PdS_x_/CNF Through the Reconfigurable Step‐Edge/Nanoparticle Interface Under Wide Potential Cycling

**Figure 3:**
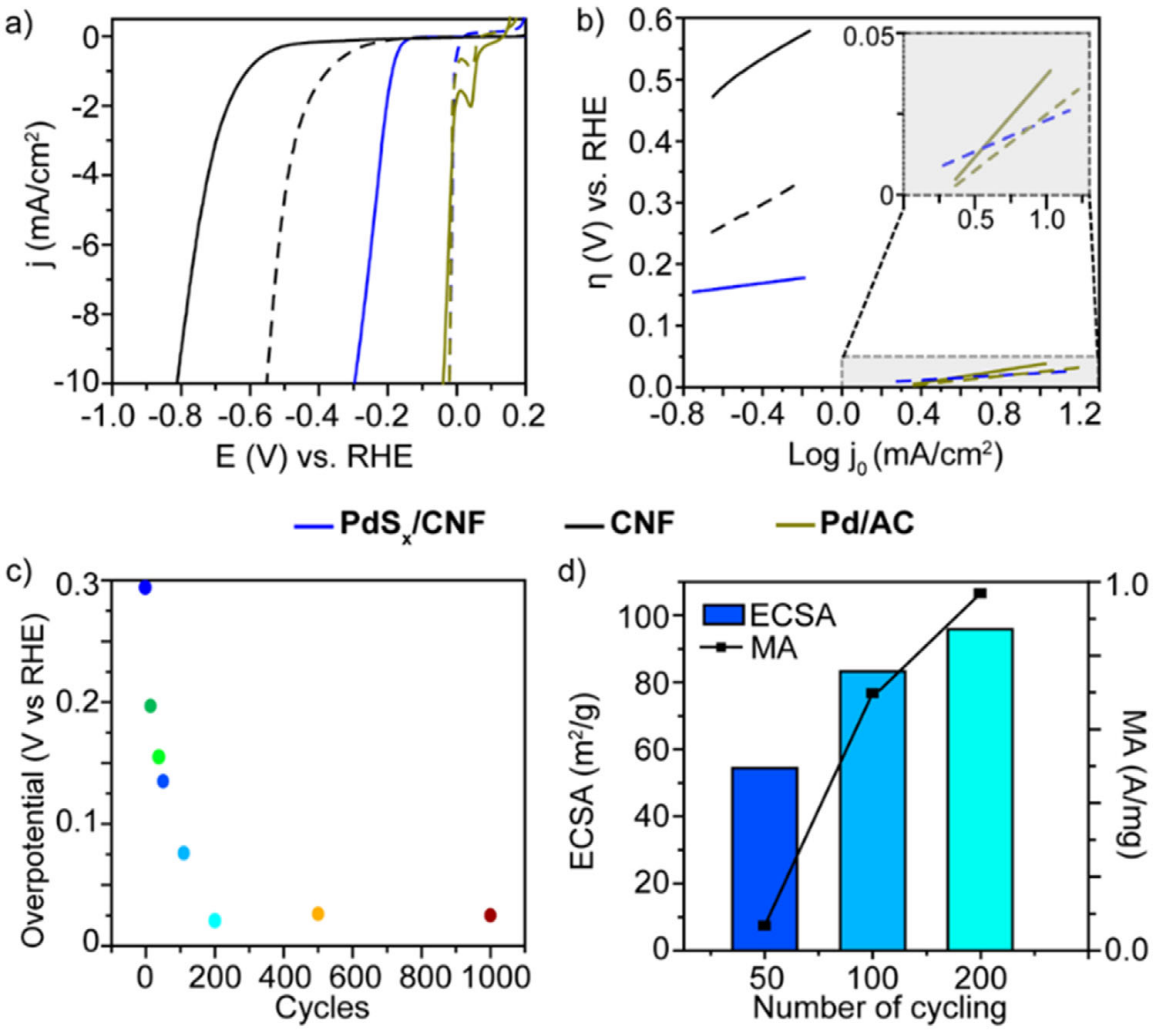
Electrochemical performance and activation of PdS_x_/CNF. a) LSV curves and b) Tafel plots of PdS_x_/CNF, CNF, and Pd/AC. Solid lines correspond to initial performance, and dashed lines after 200 potential cycles. c) Overpotential at 10 mAcm^−2^ of PdS_x_/CNF during the electrochemical activation up to 1000 potential cycles. d) Change on the electrochemical surface area (ECSA) and mass activity (MA) of PdS_x_/CNF with potential cycling. Determination of ECSA was achieved by using the peak area of hydrogen adsorption region (H_UPD_) from the CV curves (Figure S10, Supporting Information) upon cycling.^[^ ^21^ ^]^

Initial electrochemical investigations of the PdS_x_/CNF hybrid showed that the hybridization with PdS_x_ nanoparticles greatly increased their activity toward the electrochemical production of hydrogen compared to bare CNF (**Figure** **3**a; Table S4, Supporting Information). However, such improvement is still insufficient compared to the performance of the state‐of‐the‐art catalysts (Pd/AC, Figure 3a). The Tafel plot (Figure 3b) indicates very good kinetics for PdS_x_/CNF in comparison with CNF. After testing the efficiency of the material toward HER, cyclability becomes another decisive parameter. Thus, the electrochemical performance of PdS_x_/CNF toward HER was investigated after carrying out several wide potential cycles (from −0.9 to 1.2 V). As can be seen from the linear sweep voltammetry (LSV) measurements (Figure 3a; Figure S9, Supporting Information), both the onset potential and the overpotential continue to improve with the potential cycling: a decrease in the overpotential of more than 270 mV and an onset potential near to 0 mV after 200 potential cycles (Figure 3a). In fact, the Tafel slope values of the activated PdS_x_/CNF (labeled as Pd/S_x_/S‐CNF with a Pd content of 11.7 wt% as quantified by Inductively Coupled Plasma Optical Emission Spectrometry [ICP‐OES]) are very similar to those of the Pd/AC (20 wt%) benchmark catalysts (Figure 3b, Table S4, Supporting Information), revealing a Volmer‐Tafel mechanism.^[^ ^20^ ^]^ The material was tested further up to 1000 potential cycles, exhibiting extraordinary stability (Figure 3c, Table S4, Supporting Information). The HER performance improvement during potential cycling is also accompanied by an increase in electrochemical surface area (ECSA), mass activity (MA), as seen in Figure 3d, and specific activity (SA) (Figure S11, Supporting Information). An electrochemical activation (in terms of onset potential and overpotential) is also observed in the CNF material during the same potential cycling, but not to the same extent as in PdS_x_/CNF, so the overpotential of activated CNF is still far from 0 V (i.e., 552 mV). These electrochemical activations are in agreement with previous work performed in our group, in which we demonstrated that carbon nanostructures play a role in hydrogen evolution reaction.^[^ ^10^ ^]^ More specifically, the opening of graphitic step‐edges inside CNF by the electrocatalytic potential cycling. In this work, we propose a mechanism demonstrating how, under HER conditions, protons can react with C═C bonds at the curved graphitic step‐edges along the zigzag direction of the carbon sheet, leading to the reversible formation of a zigzag line of C─H bonds. Further hydrogenation subsequently causes the rupture of the curved carbon structure and the formation of flat graphene layers with hydrogen‐terminated carbon bonds in acidic media. As a result, HER activity improves, as the catalytically active sites containing edge carbon dangling bonds and residual oxygen functional groups can promote electron transfer.^[10a]^

**Figure 4:**
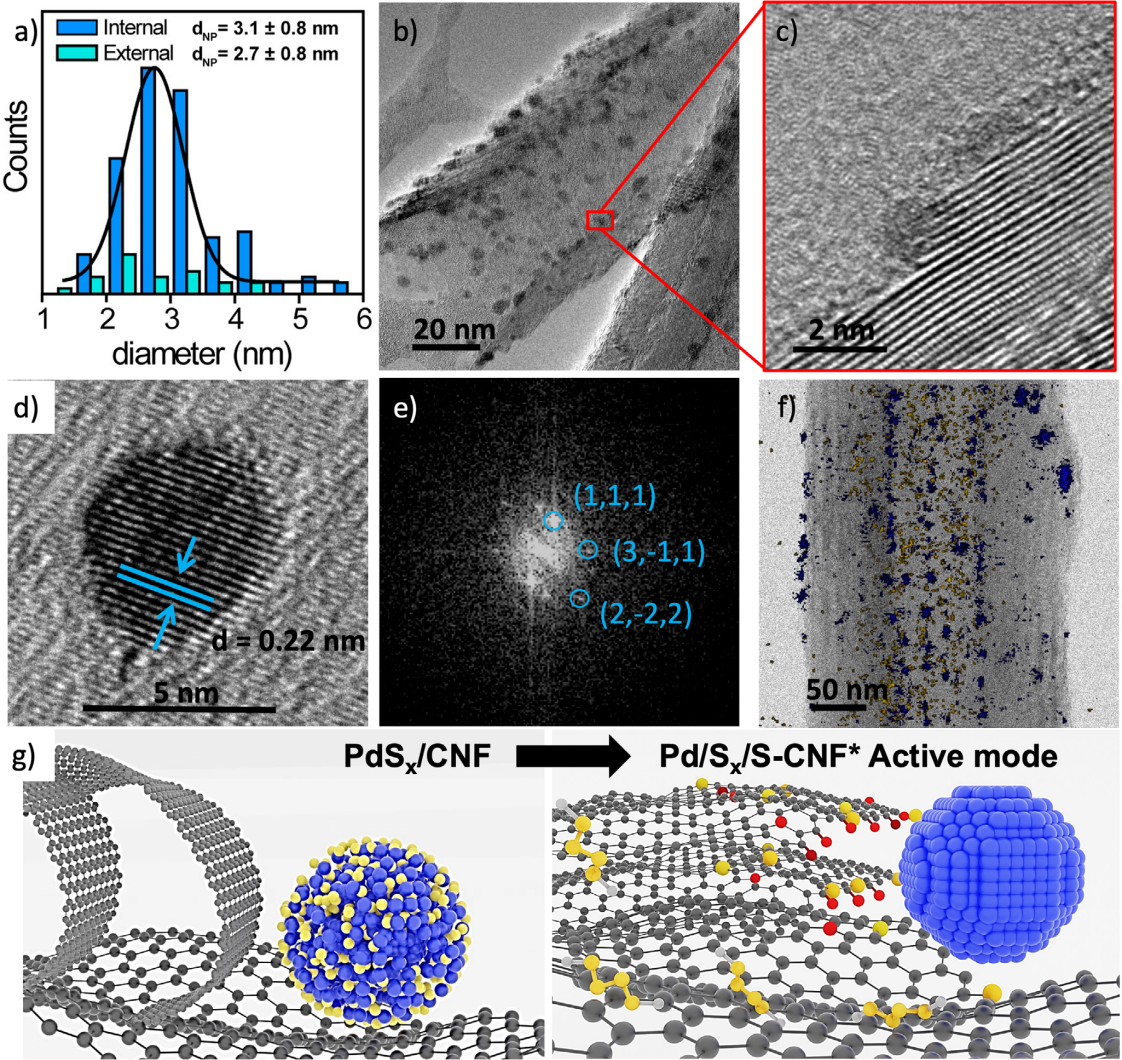
Structural characterization of Pd/S_x_/S‐CNF after electrochemical activation. a) Particle size distribution. b) HRTEM image of Pd/S_x_/S‐CNF. A high magnification HRTEM image of c) the interior of Pd/S_x_/S‐CNF showing a nanoparticle hardwire in an unfolded step‐edge and d) a Pd nanoparticle showing the interplanar distance corresponding to Pd(0) and e) FFT of (d). f) STEM‐EDS mapping image, being Pd (blue) and S (yellow). g) Schematic illustration of the activation of the material, going from PdS_x_/CNF (initial) to Pd/S_x_/S‐CNF (active mode) in which step edges are unfolded and functionalized with oxygen (red balls) and sulfur (yellow balls) at the same time that polysulfide anions and metallic palladium nanoparticles are formed.

Far more interesting about the PdS_x_/CNF material is the rise in current in the hydrogen adsorption region (H_UPD_) with potential cycling, as reflected in the LSV profiles (Figure S12, Supporting Information). This feature in the 0–50 mV region is due to the formation of different Pd‐H phases as a consequence of the hydrogen absorption by metallic Pd.^[^ ^22^ ^]^ In view of these results, it would be logical to hypothesize that during cycling activation, the chemical nature of the PdS_x_ nanoparticles changes. It is proposed that the surface of the PdS_x_ nanoparticle is electrochemically activated over a wide potential range (from −0.9 to 1.2 V), where both the reduction and oxidation of sulfur and palladium can occur. To demonstrate this hypothesis, HRTEM analysis of PdS_x_/CNF after 200 potential cycles (Pd/S_x_/S‐CNF) was performed and analyzed in detail along with STEM‐EDS. Statistical analysis of the nanoparticles exhibited smaller sizes both inside and outside compared to the initial sample (≈4 nm), with sizes of 3.1 ± 0.8 nm and 2.7 ± 0.8 nm, respectively (**Figure** **4**a). Very small nanoparticles were localized only in the interiors of CNF (less than 1 nm, Figure S13, Supporting Information), which could indicate that, during activation, palladium nanoparticles dissolve and redeposit afterward. Moreover, high‐magnification HRTEM images illustrate that step‐edges, made of folded‐graphene, known to be very sensitive to potential, unfold (Figure 4b,c). In fact, the opening of the folded‐edges would be consistent with the observed enhancement of the electrocatalytic performance of CNF during wide potential cycling, as open edges, made of oxygen‐containing groups, interact better with protons, enhancing HER. Remarkably, HRTEM also showed a clear difference in the crystallinity of the rounded‐shape palladium nanoparticles in the Pd/S_x_/S‐CNF material. While in PdS_x_/CNF the lattice fringes could not be distinguished because of the nanoparticles' amorphous nature, in the activated material (Pd/S_x_/S‐CNF) interplanar distances of 0.22 nm (corresponding to the (111) lattice plane of the face‐centered cubic (fcc) structure for Pd) were clearly observed (Figure 4d).^[^ ^23^ ^]^ The FFT in Figure 4e revealed the lattice periodicities of these palladium nanoparticles, which can be indexed in the Fm̅3m space group. The loss of sulfur from the nanoparticle during the electrochemical activation is further confirmed by STEM‐EDS mapping, as no sulfur was detected on the nanoparticles located outside CNF after 200 potential cycles (Figure 4f). This is further evidenced after 1000 potential cycles when performing EDX analysis for both confined and unconfined particles, and overlaying the EDS elemental mappings for sulfur and palladium (Figure S14, Supporting Information). It is worth noting that the average particle size of the unconfined nanoparticles slightly decreases from 2.7 ± 0.8 nm to 2.1 ± 0.7 nm as the number of potential cycles increases from 200 to 1000, while average size of confined particles remains constant (≈3.1 nm). As clearly shown in Figure S14 (Supporting Information), the detection of sulfur inside carbon nanofibers can be related to the formation of soluble polysulfides and sulfur‐terminated step‐edges, being the later induced through the reaction of the carbon dangling bonds with sulfur.^[^ ^24^ ^]^ In any case, confinement prevents the dissolution of sulfur out of the CNF.

In order to prove the importance of our protocol in the electro‐activation of PdS_x_/CNF, a potential step experiment was carried out on PdS_x_/CNF through pulses at two different potentials: between a highly oxidizing (1.2 V) and a very reducing (−0.9 V) potential. As a result, the electrocatalytic performance did not improve (Figure S15, Supporting Information). HRTEM of PdS_x_/CNF after potential step experiment showed that the internal graphitic structure is still well intact, while EDS mapping shows sulfur only where palladium nanoparticles were located (both inside and outside CNF) (Figure S16, Supporting Information). Thus, PdS_x_/CNF was not activated, as neither the folded‐edges were oxidized nor sulfide was dissolved from the nanoparticles. It can be concluded that the release of sulfur atoms accompanied by the formation of Pd(0) together with the oxidation of the open edges in CNF, is a crucial process for the electrochemical activation of PdS_x_/CNF into Pd/S_x_/S‐CNF. Due to the complex nature of the active Pd/S_x_/S‐CNF material, elucidation of mechanistic details possesses a significant challenge. However, a hypothesized route for the electro‐activation of PdS_x_/CNF into Pd/S_x_/S‐CNF is suggested based on the findings mentioned above (Figure S17, Supporting Information). First, PdS_x_/CNF, produced from thermally treating Pd@SH‐R/CNF, reveals an overall low activity toward HER due to the combination of low‐reactive PdS_X_ nanoparticles and CNF with folded step‐edges on the interior (Figure 4g). Our goal was to simultaneously activate both the amorphous PdS_x_ and CNF by electrochemical means, dissolving the nanoparticles into their individual components (Pd and S), together with the unfolding of the step‐edges in CNF. To do so, a wide potential window was required, where the potential is positive enough to oxidize folded step‐edges (i.e, 1.2 V) and negative enough (i.e., −0.9 V) to induce the dissolution of NPs by the formation of polysulfides. We take advantage of the fact that confinement will hinder the leaching of S and Pd atoms, and subsequent loss into the bulk electrolyte during nanoparticle reconfiguration. Hence, it is proposed that CNF step‐edges become unfolded at positive potential during potential cycling, leaving the interior with oxidized functional groups and carbon dangling bonds that are more reactive. This phenomenon has been previously observed in a study where tiny Pd NPs were supported in graphitized CNF, leading to an opening up of the step edges under similar conditions.^[10a]^ Conversely, the release of sulfur atoms from amorphous PdS_x_ at negative potential will facilitate the migration and reaction of sulfur with itself (forming series of polysulfides), as well as with defects (carboxyl, hydroxyl groups) on the carbon surface and the recently unfolded step‐edges (C dangling bonds), forming C─S bonds (Figure 4g). To verify that the reaction between sulfur and the carbon surface would lead to an electroactive material, the activity of a S‐CNF control material was evaluated.

As seen in Figure S18 (Supporting Information), S‐CNF exhibits higher activity than CNF, with the overpotential of S‐CNF being similar to that of CNF with unfolded step‐edges induced by electrochemical means (510 and 540 mV, respectively). Simultaneously, the release of sulfur atoms from amorphous PdS_x_ favors the recrystallization of PdS_x_ into Pd. This transformation of PdS_x_ into Pd is supported by HRTEM measurements, FFT analysis, and the rise in the H_UPD_ peak observed in LSV polarization curves after 200 potential cycles. Note that the release of sulfur atoms from the nanoparticles is accompanied by Pd^2+^, which is subsequently deposited at negative potential inside CNF, leading to the formation of small NPs (i.e., < 1 nm, Figure S13, Supporting Information). It is proposed that such in situ reconfiguration of PdS_x_ nanoparticles into Pd, along with the doping of carbon edges and the formation of polysulfides (Figure 4g), enhances the electron transfer, resulting in an excellent material with significantly improved HER activity. Chronoamperometry measurements at extreme potentials (i.e., −0.5 and −0.9 V) further demonstrate the catalyst's operational stability (Figure S19a,b, Supporting Information), with sustained activity confirmed by the characteristic hydrogen desorption peak (H_UPD_) of Pd observed in the corresponding CV curves (Figure S19c, Supporting Information).

### 2.3 Electrochemical Switching Ability of a Reconfigurable Step‐Edge/Nanoparticle Interface

**Figure 5:**
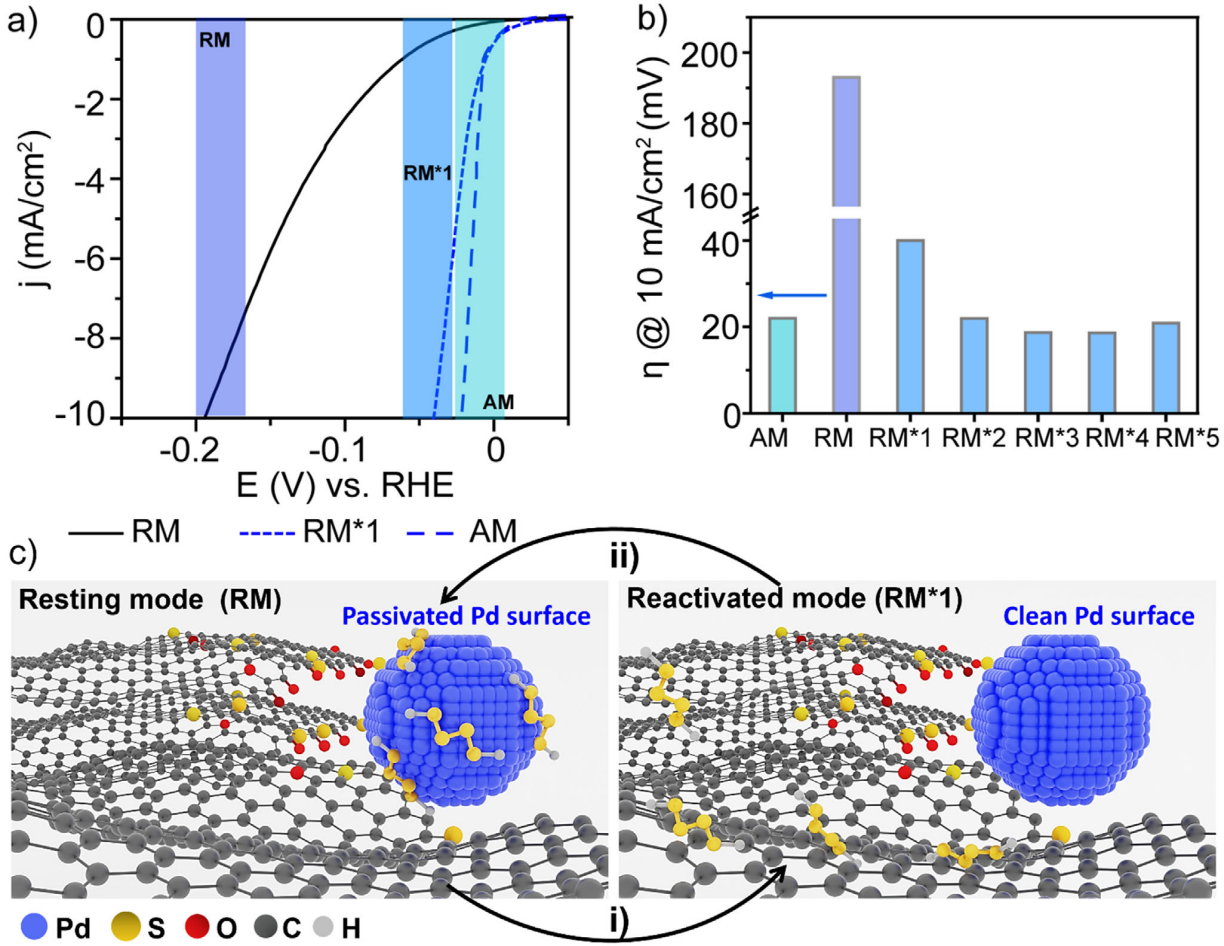
Electrochemical switching effect. a) HER‐LSV curves related to the open‐circuit potential experiment. b) switching capacity of Pd/S_x_/S‐CNF* material, which initially transitions from active mode (AM) to resting mode (RM) after undergoing 750 CV within −0.2 to 0.4 V range. The material is reactivated through wide window potential CV (−0.9 V to 1.2 V), reaching RM*1. The process is repeated five times, where the material alternates between active and multiple resting modes (RM*1–5) during each cycle through wide window potential (−0.9 V to 1.2 V). c) Scheme for process of the switching between the activation and the resting mode of Pd/S_x_/S‐CNF, where i) is the wide window potential CV (from −0.9 V to 1.2 V) and ii) is a OCP experiment and/or a CV in the range of −0.2 to 0.4 V. AM, RM and RM* stands for active mode, resting mode and reactivated mode of Pd/S_x_/S‐CNF, respectively.

As discussed, the migration of sulfur atoms from the assembled nanoparticles results in an electrode (Pd/S_x_/S‐CNF) with extraordinary electrochemical kinetics and effectiveness toward HER (Table S5, Supporting Information). In the context of commercial applications, the switching ability and speed of a system are crucial for device operation. A key aspect of this work is the development of an electrochemical procedure to fine‐tune the electrochemical properties of the electrode. We will refer to the Pd/S_x_/S‐CNF material as the “active mode” (AM). However, applying the appropriate potential does not necessarily ensure that its electrochemical activity toward the HER remains permanent. In fact, a significant decrease in HER activity is observed when Pd/S_x_/S‐CNF is oxidized at a potential where polysulfides are not stable (i.e., higher than −0.4 V, S +2e^−^ → S^2−^ at E_0_ = −0.4 V), either by applying small window potential cycles (from −0.2 to 0.4 V) (Figure S20c, Supporting Information) or by measuring the open circuit potential (OCP) (**Figures** **5**a and S20a,b, Supporting Information). This leads to the emergence of a much less active state, which we refer to as the “resting mode” (RM) (Figure 5a). In this state, the onset potential and overpotential increased by 22 and 193 mV, respectively. Additionally, Tafel slope values increased from 34 to 113 mV dec^−1^, indicating slower kinetics in the resting mode (Table S6, Supporting Information).

The cyclic voltammogram features of the material in the resting mode revealed no peak of H_UPD_, indicating that the electroactive sites of crystalline palladium nanoparticles are passivated (Figure S21, Supporting Information). This passivation obstructs Pd active sites, hindering the adsorption of protons on the surface and thereby reducing hydrogen production. It is proposed that the soluble polysulfides, generated during the electrochemical activation, are oxidized to sulfur under these electrochemical conditions and tend to block the palladium surface (Figure 5c(ii)).^[^ ^25^ ^]^ This poisoning effect is consistent with previous studies reporting the loss of catalytic activity in metallic palladium due to the strong interaction of insulating sulfur with its surface.^[^ ^26^ ^]^

To confirm that the formation of a resting mode (RM) is related to the passivation of palladium nanoparticles by oxidized polysulfides, a composite consisting of palladium nanoparticles and polysulfide chains inside carbon nanofibers (Pd/polyS@CNF) was synthesized as a control experiment. The STEM‐EDX mapping of Pd/polyS@CNF confirms the presence of palladium and sulfur atoms within the cavities of CNF (Figure S22, Supporting Information), while XRD and Raman confirm the presence of polysulfide. Interestingly, the cyclic voltammograms of Pd/polyS@CNF (Figure S23, Supporting Information) resemble those of Pd/S_x_/S‐CNF in the resting mode (RM), as no peak in the hydrogen adsorption region (H_UPD_) is observed, indicating that the surface of palladium nanoparticles is inactive due to the adsorption of sulfur (resulting from polysulfide oxidation). Furthermore, the Tafel slopes of Pd/polyS@CNF and Pd/S_x_/S‐CNF in the resting mode (RM) are similar (i.e., 100 and 113 mV dec^−1^, respectively) (Table S7, Supporting Information), further supporting the hypothesis that the electrochemical oxidation of polysulfides can block the surface of palladium nanoparticles at positive potential, thereby decreasing their electrochemical activity toward HER.

Pd/S_x_/S‐CNF in the resting mode (RM) can be reactivated by performing a single potential cycle in a wide‐window potential range (i.e., −0.9 V to 1.2 V), leading to a reactivated mode (RM*) with electrochemical values very close to those of the active mode (AM) (Figure 5a; Table S6, Supporting Information). Both RM* and AM show similar catalytic activity, but they originate differently– RM* emerges after reactivating a resting state (RM), while AM is achieved through direct activation of the initial inactive PdS_x_/CNF material.

The ECSA, MA, and SA values for RM* were obtained and compared with those of the AM (Pd/S_x_/S‐CNF) and the Pd/AC benchmark (20 wt.%), as depicted in Table S8 (Supporting Information). These results demonstrate that the material can be significantly reactivated, along with a more effective use of palladium, improving both performance and cost‐effectiveness on a per‐mass‐of‐Pd basis. This is a consequence of the electrochemical reversibility of sulfur, which enables the formation of polysulfides species under these electrochemical conditions (Figure 5c(i)).^[^ ^27^ ^]^

The switching effect observed in this work surpasses current studies where the passivation of electrochemical active nanoparticles is irreversible. For example, Li et al, demonstrated that the electrochemical oxidation of RuNP irreversibly diminishes the adsorption of hydrogen atoms on its surface, making subsequent reactivation of the material impossible.^[^ ^28^ ^]^ Thus, the role of sulfur in the electrochemical switching exhibited by Pd/S_x_/S‐CNF* material is believed to be crucial, as this phenomenon has not been observed in comparable materials. For instance, palladium nanoparticles confined within CNF (i.e., PdNP@GNF, similar to PdS_x_/CNF material but without sulfur) did not show the same decrease in HER activity as Pd/S_x_/S‐CNF during potential cycling in the same potential range (i.e., −0.2 V to 0.4 V, Figure S18, Supporting Information).^[10a]^ This confirms that sulfur is essential, as it plays a pivotal role in achieving electrochemical switching ability. Figure S18 (Supporting Information) presents a representative illustration and comparison between Pd/S_x_/S‐CNF and its controls (PdNP@GNF, CNF, polyS@CNF, and S‐CNF).

The long‐term stability of Pd/S_x_/S‐CNF was also evaluated, demonstrating its ability to undergo repeated switching, as shown in Figure 5b. Initially, the Pd/S_x_/S‐CNF active mode (AM) material transitions into the resting mode (RM) after 750 small‐window potential cycles. Subsequently, the material is reactivated through a single potential cycle in a wide potential window, returning to a reactivated state (RM*1) with an overpotential of 44 mV. This switching process occurs repeatedly over five cycles, with the material transitioning between resting mode (RM) and multiple reactivated modes (RM*1–5).

In our work, we demonstrate for the first time the possibility of regenerating the surface of an electroactive material by using sulfur as a reversible mediator, enabling the deliberate tuning of its electrocatalytic performance through the selective activation and deactivation of the adsorption of protons on the surface of palladium. Overall, it is important to highlight that this activation and subsequent switching effect is made possible by the confinement within the carbon structure, preventing the loss of sulfur and palladium from the electrode, as well as by the electrochemical reversibility of the sulfide/sulfur redox couple. As discussed, the reduction and oxidation of carbon play a crucial role in the electrochemical performance of Pd/S_x_/S‐CNF.

## 3 Conclusion

The synthesis of a novel hybrid electrocatalyst material consisting of amorphous palladium sulfide (PdS_x_) nanoparticles hardwired in hollow graphitized CNF has been reported. The disordered structure of PdS_x_ combined with the highly reactive internal carbon nanostructure, enables the surface reconfiguration of the deactivated electrocatalyst through potential cycling, resulting in an activated Pd/S_x_/S‐CNF with superior electrocatalytic performance toward HER.

More interestingly, the confinement within the CNF extends the lifespan of the hybrid and facilitates the retention of sulfur, which plays a crucial role in modulating the electrochemical performance of the activated Pd/S_x_/S‐CNF. Indeed, sulfur species act as a mediators, enabling the transition from an active mode (AM) to a less active resting mode (RM), followed by subsequent recovery of activity (reactivated mode, RM*), through reversible interactions either with the carbon support or the catalytic metal center.

Overall, our unique discovery introduces a novel methodology to control the performance of metal nanoparticles for catalytic reactions and, more importantly, enhancing their lifespan.

## Conflict of Interest

The authors declare no conflict of interest.

## Supporting information



Supporting Information

## Data Availability

The data that support the findings of this study are available in the Supporting Information of this article.

## References

[1] a) T. J. Meyer , Nature 2008, 451, 778; 18273008 10.1038/451778a

[2] a) Z. W. Seh , J. Kibsgaard , C. F. Dickens , I. Chorkendorff , J. K. Nørskov , T. F. Jaramillo , Science 2017, 355, aad4998; 10.1126/science.aad4998 28082532

[3] a) V. Celorrio , D. Sebastián , L. Calvillo , A. B. García , D. J. Fermin , M. J. Lázaro , Int. J. Hydrogen Energy 2016, 41, 19570;

[4] a) J.‐h. Myung , D. Neagu , D. N. Miller , J. T. S. Irvine , Nature 2016, 537, 528; 27548878 10.1038/nature19090

[5] a) F. Liu , L. Zhang , L. Wang , F. Cheng , Electrochem. Energy Rev. 2021, 4, 146;

[6] a) L. Liu , A. Corma , Chem. Rev. 2018, 118, 4981; 29658707 10.1021/acs.chemrev.7b00776 PMC6061779

[7] a) L. He , W. Zhang , Q. Mo , W. Huang , L. Yang , Q. Gao , Angew. Chem., Int. Ed. 2020, 59, 3544; 10.1002/anie.201914752 31880061

[8] a) K. Guo , Y. Wang , J. Huang , M. Lu , H. Li , Y. Peng , P. Xi , H. Zhang , J. Huang , S. Lu , C. Xu , ACS Catal. 2021, 11, 8174;

[9] a) X. Wu , Z. Wang , K. Chen , Z. Li , B. Hu , L. Wang , M. Wu , ACS Appl. Mater. Interfaces 2021, 13, 22448; 33950664 10.1021/acsami.1c03350

[10] a) M. Aygün , M. Guillen‐Soler , J. M. Vila‐Fungueiriño , A. Kurtoglu , T. W. Chamberlain , A. N. Khlobystov , M. Del Carmen Gimenez‐Lopez , ChemSusChem 2021, 14, 4973; 34132044 10.1002/cssc.202101236 PMC9292725

[11] a) A. La Torre , M. del Carmen Gimenez‐Lopez , M. W. Fay , C. H. Lucas , P. D. Brown , A. N. Khlobystov , Small 2015, 11, 2756; 25689488 10.1002/smll.201402807

[12] a) M. Brust , M. Walker , D. Bethell , D. J. Schiffrin , R. Whyman , J. Chem. Soc., Chem. Commun. 1994, 7, 801;

[13] A. La Torre , M. d. C. Giménez‐López , M. W. Fay , G. A. Rance , W. A. Solomonsz , T. W. Chamberlain , P. D. Brown , A. N. Khlobystov , ACS Nano 2012, 6, 2000. 22356571 10.1021/nn300400z

[14] G. Corthey , J. A. Olmos‐Asar , G. Casillas , M. M. Mariscal , S. Mejía‐Rosales , J. C. Azcárate , E. Larios , M. José‐Yacamán , R. C. Salvarezza , M. H. Fonticelli , J. Phys. Chem. C 2014, 118, 24641.

[15] J. C. Azcárate , G. Corthey , E. Pensa , C. Vericat , M. H. Fonticelli , R. C. Salvarezza , P. Carro , J. Phys. Chem. Lett. 2013, 4, 3127.

[16] J. C. Love , D. B. Wolfe , R. Haasch , M. L. Chabinyc , K. E. Paul , G. M. Whitesides , R. G. Nuzzo , J. Am. Chem. Soc. 2003, 125, 2597. 12603148 10.1021/ja028692+

[17] S. Kumar , S. Soni , W. Danowski , C. L. F. Van Beek , B. L. Feringa , P. Rudolf , R. C. Chiechi , J. Am. Chem. Soc. 2020, 142, 15075. 32786759 10.1021/jacs.0c06508 PMC7472521

[18] a) W. Lu , B. Wang , K. Wang , X. Wang , J. G. Hou , Langmuir 2003, 19, 5887;

[19] L. C. Chen , Q. Peng , H. Yu , H. J. Pang , B. B. Jiang , L. Su , X. Shi , L. D. Chen , X. J. Chen , J. Alloys Compd. 2019, 798, 484.

[20] Y. Jiao , Y. Zheng , M. Jaroniec , S. Z. Qiao , Chem. Soc. Rev. 2015, 44, 2060. 25672249 10.1039/c4cs00470a

[21] C. Wei , R. R. Rao , J. Peng , B. Huang , I. E. L. Stephens , M. Risch , Z. J. Xu , Y. Shao‐Horn , Adv. Mater. 2019, 31, 1806296. 10.1002/adma.201806296 30656754

[22] S. Hu , J. Noborikawa , J. Haan , L. Scudiero , S. Ha , ECS Trans. 2014, 64, 1113.

[23] G. Han , Q. Jiang , W. Ye , C. Liu , X. Wang , Sci. Rep. 2019, 9, 7300. 31086221 10.1038/s41598-019-43840-0 PMC6514013

[24] J. Li , S. Yang , W. Wu , H. Jiang , Org. Chem. Front. 2020, 7, 1395.

[25] a) N. Sergienko , E. Irtem , O. Gutierrez , J. Radjenovic , J. Hazard. Mater. 2019, 375, 19; 31035182 10.1016/j.jhazmat.2019.04.033

[26] a) J. Liu , K. Kimmel , K. Dao , Y. Liu , M. Qi , Org. Process Res. Dev. 2018, 22, 111;

[27] a) S. Huang , Y. V. Lim , X. Zhang , Y. Wang , Y. Zheng , D. Kong , M. Ding , S. A. Yang , H. Y. Yang , Nano Energy 2018, 51, 340;

[28] J. Li , S. Ghoshal , M. K. Bates , T. E. Miller , V. Davies , E. Stavitski , K. Attenkofer , S. Mukerjee , Z. F. Ma , Q. Jia , Angew. Chem., Int. Ed. 2017, 56, 15594. 10.1002/anie.201708484 29044864

