# Supplementary material for: Adaptive Catalytic Nanointerfaces for Controlled Hydrogen Evolution: an in Situ Electrochemical Approach
Source: Adv Sci (Weinh). 2025 May 23;12(30):e05104. doi: 10.1002/advs.202505104 (PMC12376495; doi:10.1002/advs.202505104)
Supplement: Supplementary file 1 — Supporting Information [file ADVS-12-e05104-s001.pdf]

ADVANCED SCIENCE
Open Access

Supporting Information

for *Adv. Sci.*, DOI 10.1002/advs.202505104

Adaptive Catalytic Nanointerfaces for Controlled Hydrogen Evolution: an in Situ Electrochemical Approach

*Carlos Herreros-Lucas, Melanie Guillén-Soler, Lucía Vizcaíno-Anaya, Glen Murray, Mehtap Aygün, José Manuel Vila-Fungueiriño and María del Carmen Giménez-López**

Supporting Information

Adaptive Catalytic Nanointerfaces for Controlled Hydrogen Evolution: An In Situ Electrochemical Approach

Carlos Herreros-Lucas, [a] Melanie Guillén-Soler, [a] Lucía Vizcaíno-Anaya, [a] Glen Murray, [b] Mehtap Aygün, [a] José Manuel Vila-Fungueiriño, [a] and María del Carmen Giménez-López*[a,b]

[a] Dr. C. Herreros-Lucas, Dr. M. Guillén-Soler, L. Vizcaíno-Anaya, Dr. M. Aygün, Dr. J. M. Vila-Fungueiriño, Prof. M. C. Giménez-López
Centro Singular de Investigación en Química Biolóxica e Materiais Moleculares (CiQUS), Universidade de Santiago de Compostela, 15782 Santiago de Compostela, Spain.
Email: maria.gimenez.lopez@usc.es

[b] Dr. G. Murray, Prof. M. C. Giménez-López
School of Chemistry, The University of Nottingham, University Park, Nottingham, NG7 2RD, UK

Materials and methods

CNF (PR24) was purchased from Pyrograf Products Inc (USA). Palladium, nominally 20%wt. on activated carbon black was purchased from Alfa Aesar (UK), respectively. All other reagents and solvents were purchased from Sigma Aldrich (Spain) and used without any further purification. TEM was performed on a JEOL JEM F200 microscope equipped with a cold field-emission gun (Cold-FEG) operated at 200 kV with an ultra-high-resolution pole piece. TEM images were acquired using a Gatan OneView camera. Energy Dispersive X-ray Spectroscopy (EDS) was performed with a Centurio Large Angle Silicon Drift Detector (SDD) with a detection area of 100 mm^2. TEM specimens were prepared by casting several drops of a suspension of the carbon material in hexane onto a nickel or copper-grid mounted "holey" carbon film before drying under a stream of nitrogen. TGA was carried out on an SDT Q-600 TA instrument over the range of 25-1000 °C in air with a scan rate of 5 °C/min. The Raman spectra were performed with RENISHAW Raman microscope with laser Ion Ar (514 nm). XPS analysis of the samples was performed using a Thermo Scientific K-Alpha ESCA/XPS instrument equipped with Al Kα monochromatized radiation (1486.6 eV). To guarantee a homogeneous surface conductivity, a charge compensation (electron flood) gun was used to minimize surface charging. Surface charge compensation was performed by using both a low energy flood gun (electrons in the range 0 to 14 eV) and a low energy Argon ion gun. The XPS measurements were carried out using monochromatic Al-Ka radiation (1486.6 eV). Photoelectrons were collected from a take-off angle of 90° relative to the sample surface. The measurement was done in a Constant Analyser Energy mode (CAE) with a 100 eV pass energy for survey spectra and 30eV pass energy for high resolution spectra. The high-resolution XPS spectra of S 2p and Pd 3d core levels were deconvoluted assuming a Gaussian:Lorentzian (70%:30%) line shape and a Shirley background. The S 2p core levels were fitted using two pairs of spin−orbit split components (S($2p_{3/2}$) and S($2p_{1/2}$)) and a splitting energy fixed at 1.16 eV. The line shapes of the high-resolution Pd 3d core level spectra were also fitted using two pairs of spin−orbit split components (Pd($3d_{5/2}$) and Pd($3d_{3/2}$)) and a splitting energy fixed at 5.26 eV. Pd content was quantified by ICP-OES after acid digestion using microwave-assisted decomposition.

Synthesis of preformed palladium nanoparticles (Pd@SH-R)

The synthesis of dodecanethiolate-stabilized PdNP (Pd@SH-R) was performed using a modified Brust–Schiffrin protocol. To an aqueous solution of the metal salt (0.2 mmol, K_2PdCl_4) in deionized water (30 mL), a solution containing tetraoctylammonium bromide (0.4 mmol, 223 mg) in toluene (60 mL) was added and vigorously stirred at room temperature for 10 min. Dodecanethiol (0.2 mmol, 50 µL) in toluene (10 mL) was then added to the mixture that was vigorously stirred at room temperature for another 10 min. To the reaction mixture was added a further solution containing sodium borohydride (2.0 mmol, 75.6 mg) in deionized water (30 mL). The resulting black mixture was vigorously stirred at room temperature for 20 hours. The biphasic mixture was then separated, retaining the organic layer which was washed with water (3 x 100 mL) and dried over anhydrous sodium sulfate. This was concentrated (*c.a.* 5 mL) under vacuum and the product precipitated from solution by addition of ethanol (350 mL) and storing at -30˚C for 24 hours. The resulting precipitate was filtered through a 0.2 µm pore size PTFE membrane, the solid washed with ethanol (200 mL), acetone (200 mL) and finally dried under vacuum to yield a brown solid product (56.7 mg Pd@SH-R).

Synthesis of milled carbon nanofibers (CNF)

Milled carbon nanofibers (CNF) were produced by mechanical ball milling using a Retsch MM400 ball mill instrument (600 rpm). In a typical experiment, 50 mg of CNF (Pyrograh, PR24) were placed into a stainless-steel container (5mL) with a stainless-steel ball (10 mm diameter) and milled in air for 180 min at 600 rpm (98% yield). No further purifications steps were required.

Synthesis of Pd@SH-R/CNF

Initially, CNF (10 mg) were dispersed in hexane (10 mL) by sonication for 10 minutes and added dropwise to a stirring suspension of Pd@SH-R (20 mg) in hexane. The resulting dark suspension was sonicated for 15 min. Subsequently, it was filtered through a 0.2 µm pore size PTFE membrane, the solid washed with hexane (200 mL), acetone (200 mL) and finally dried under vacuum.

Synthesis of PdS_x/CNF

The composite (10 mg, Pd@SH-R/CNF) was sealed under vacuum (10^{-5} mbar) in a Pyrex tube and placed in a pre-heated oven (300°C) for 2 hours. Once the material cooled down, the heated PdS_x/CNF composite was sonicated for 15 minutes in hexane (50mL) and filtered through a 0.2 µm pore size PTFE membrane. The solid was further washed with hexane (100 mL), acetone (200 mL) and finally dried under vacuum.

Synthesis of PdS_x/CNF-pristine

Preformed nanoparticles in pristine carbon nanofibers (Pd@SH-R/CNF-pristine) was also sealed under vacuum (10^{-5} mbar) in a Pyrex tube and placed in a pre-heated oven at 300°C for 2 hours.

Synthesis of $(CS_x)_n$-CNF intermediate

Polysulfide inside carbon nanofibers (polyS@CNF) and sulfur-doped carbon nanofibers (S-CNF) were produced using the same intermediate which was recently reported by Zhang et al. [1]. Initially, elemental sulfur (8 mmol, 0.268g) and sodium sulfide (6 mmol, 0.5g) were introduced in a 50 mL round bottom flask containing 20 mL of a mixture of water and methanol (1:1) and stirred at room temperature until an orange color appeared. Separately, ball-milled carbon nanofibers (50 mg, CNF) were dispersed in 20 mL of a mixture of water and methanol (1:1) by sonication for 15 minutes. Then, the polysulfide reaction mixture was added in small portions (1 mL) to the dispersion of CNF while sonication. The reaction was sonicated for 2 hours and vacuum dried to remove the solvent afterwards. A solution of 0.1 mL of hexachlorobutadiene in 3.5mL of NMP was added dropwise to the residue previously dispersed in 2 mL N-methyl pyrrolidone (NMP). The mixture was sonicated for 2 hours. Finally, the product was filtered through PTFE membrane and washed with water, methanol, and acetone several times and then dried in vacuum for 24 hours at 60 °C.

- **Synthesis of polyS@CNF:**

$(CS_x)_n$-CNF intermediate material (150 mg) was put in a vessel of alumina and place on the center of the furnace and the programing furnace temperatures was as follows: furnace temperature was raised from room temperature to 60°C and kept for 30 minutes, then raised to 380°C with a ramp of 5°C/min and kept for 1 hour. Then, the furnace was let to cool down to room temperature.

- **Synthesis of S-CNF:**

$(CS_x)_n$-CNF intermediate material (150 mg) was put in a vessel of alumina and place on the center of the furnace and the programing furnace temperatures was as follows: furnace temperature was raised from room temperature to 60°C and kept for 30 minutes, then raised to 380°C with a ramp of 5°C/min and kept for 1 hour. Then, the temperature was raised to 600°C with a 10 °C/min ramp and held only for one minute. Finally, the furnace was let to cool down to room temperature.

Synthesis of Pd/polyS@CNF

To introduce palladium nanoparticles into **polyS@CNF,** we followed a procedure reported in ref [2]. **PolyS@CNF** (15 mg) was dispersed in $CHCl_3$ (40 mL) under sonication for 10 minutes. A solution of $Pd_2dba_3.CHCl_3$ (9.3 mg) in $CHCl_3$ (15 mL) was then slowly added to the polyS@CNF dispersion in small portions (0.5 mL) whilst being treated with ultrasonic waves. The mixture was further sonicated for 10 min. It was then stirred at 40 ° C for 1 day until the supernatant solution became colorless. **Pd/polyS@CNF** was then separated from the reaction mixture by filtration and washed repeatedly with acetone (20 mL) using a 0.2 μm PTFE membrane filter to remove the free dba and obtain the final material as a black powder. The amount (mg) of $Pd_2dba_3.CHCl_3$ added was the estimated to obtain approximately 12% of Pd (%wt) in the final material, which was calculated according to the %wt of carbon in the Sulfur-CNF composite.

Electrochemical measurements

Electrochemical experiments were carried out on a computer-controlled potentiostat (Autolab 302N) at room temperature using a conventional three-electrode cell with 0.1M $HClO_4$ aqueous solution as electrolyte. A carbon rod and a reversible hydrogen reference electrode (RHE) were used as the counter and reference electrode, respectively. For the working electrode, the catalyst material was dispersed in hexane (2 mg/mL) by sonication for 15 minutes and 14 μ L of the suspension were dropped-casted onto glassy carbon working electrodes of 5 mm in diameter. Subsequently, 20 μ L of Nafion solution in water were added and left to dry under air. All the electrochemical measurements were corrected by the ohmic potential drop (iR) losses from the solution resistance. Characteristic cyclic voltammetry (CV) measurements were taken between 0 and 1V at a scan rate of 10 mV/s, linear sweep voltammetry (LSV) for hydrogen evolution was performed between 0.2V and -0.9V at a scan rate of 10mV/s. In situ activation experiments through CVs were performed with an upper and lower potential of 1.2V and -0.9V, respectively, at a scan rate of 50mV/s starting at an applied potential of 0V. For the potential step experiments, the potential was alternated from -0.9V (1 s) to 1.2V (1 s) for a total duration of 1300 s.

The assessment of electrocatalyst materials in this study was based on several criteria including:

Onset potential (E_{onset}), which refers to the potential at which the reaction commences and current begins to increase. A lower E_{onset} indicates greater efficiency of the catalyst.

Overpotential at 10 mA/cm^2 of current density (η @10mA/cm^2), was evaluated by determining the potential required for producing a specific level of current density (j = **10mA/cm^2**).

Tafel slopes, represented changes in potential necessary to amplify reaction rates tenfold - often utilized for studying kinetics wherein lower values indicate faster reactions.

Slopes were calculated from the slope of the linear fitting in the polarization curves using the Tafel equation simplified from the Butler-Volmer equation:

$$\eta_a = \frac{0.059}{(1-\alpha_c)n} log(j_0) - \frac{0.059}{(1-\alpha_c)n} log(j) \quad \text{at 25 °C.}$$

Here η is the overpotential (η = E_0-E, i.e. the difference between the applied potential (E) and the equilibrium potential (E_0) *vs* RHE, which is 0 V for HER in acid), J_0 is the exchange current density at 0 V overpotential. This equation can be simplified to:

$$\eta = a + b\ log(j)$$

It indicates a linear relationship between the overpotential (η) and $\log(j)$ and thus a slope b is generated and described as the Tafel slope when the overpotential is plotted as a function of $\log(j)$.

Electrochemically active surface area (ECSA) is the active area of the electrode material that is accessible to the electrolyte for charge transfer. ECSA values may change after electrode operation, according to the exposure of the active sites.

ECSA values were obtained with an integration of the peak area of hydrogen desorption region (H_{UPD}) by subtracting the double-layer charging currents via cyclic voltammetry method.

$$Q_H[C] = \frac{Peak\ area\ [A \cdot V]}{Scan\ rate\ [\frac{V}{s}]}$$

$$ECSA\left[\frac{m^2}{g}\right] = \frac{Q_H[C]}{C\left[\frac{\mu C}{cm^2}\right] * L_{metal}\left[\frac{mg_{metal}}{cm^2}\right] * A_g\ [cm^2]} * 10^5$$

---

where, Q_H is the total charge obtained from CV; L_{metal} is the loading of metal catalyst on 1 cm^2 of glassy carbon electrode; C is the charge required to reduce a monolayer of protons on the metal (240 $\mu C/cm^2$ for Pd) and A_g is the geometric surface area of the glassy carbon working electrode.

The **mass activity** (MA) of the catalyst is calculated by the followed equation:

$$MA[A/mg] = \frac{r_f i_k}{L_{Pd}}$$

The roughness factor (r_f) is calculated from the equation below. The ratio between the real metal surface area A_{real} measured from CV and the geometric area of the electrode A_{geo} obtained from the surface area of the working electrode is equal to 0.196 cm^2.

$$r_f = \frac{A_{real}}{A_{geo}} = \frac{Q_H(C)}{\frac{240(\frac{\mu C}{cm^2})}{0,196\ (cm^2)}}$$

The **specific activity** (SA) for the current per unit surface area of catalyst was obtained by the following formula:

$$SA[mA/cm^2] = \frac{MA\left[\frac{A}{mg}\right] x\ 100}{ECSA\ [m^2/g]}$$

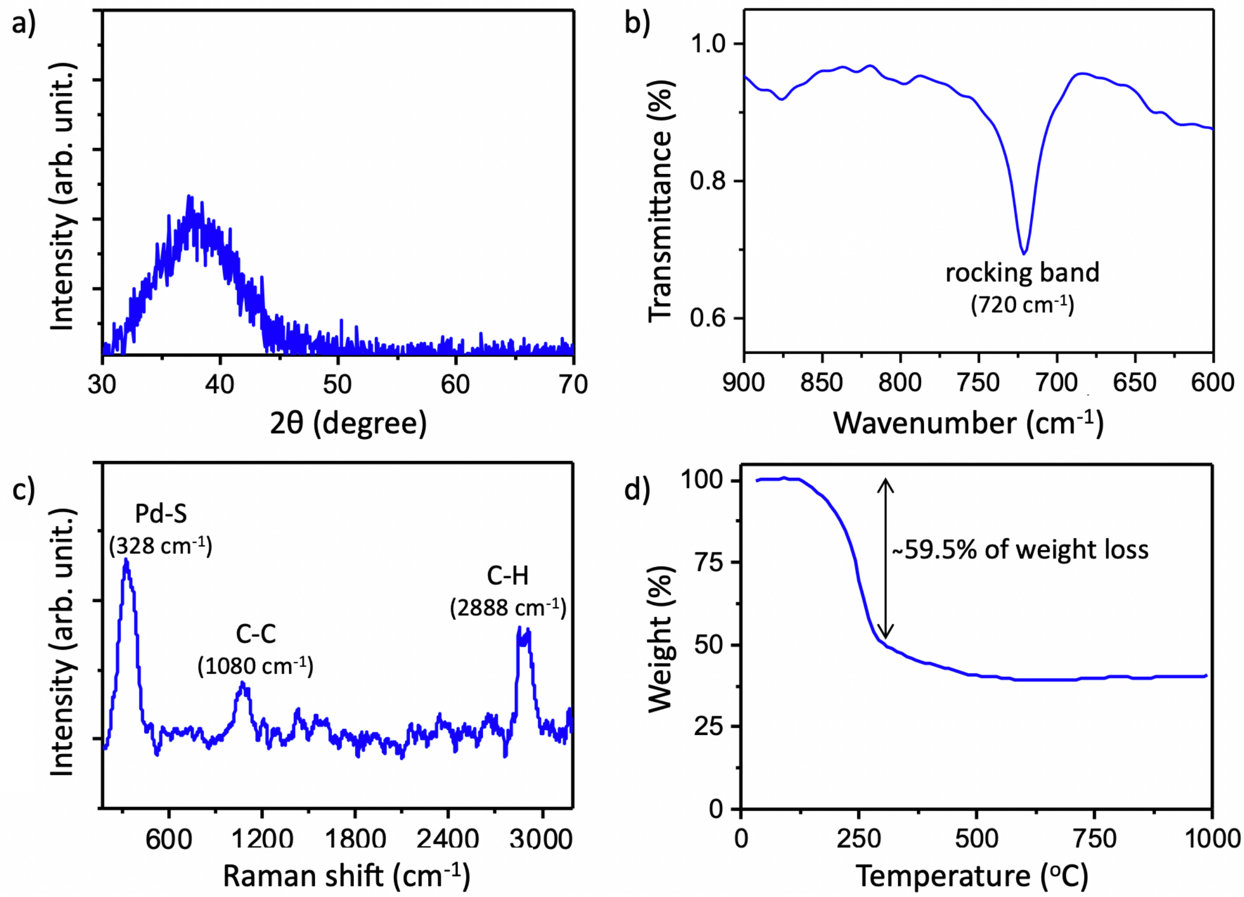

Supplementary Figure 1. (a) Powder X-ray diffraction (XRD) measurements, **(b)** Fourier Transform Infrared (FTIR) spectra, **(c)** Raman spectra and **(d)** Thermal gravimetric analysis (TGA) at 5˚C/min in air of preformed Pd@SH-R nanoparticles. A broad peak in XRD measurements indicates the small size of preformed Pd nanoparticles. IR and Raman analysis confirm the presence of dodecanethiol interacting with palladium: peak at 720 cm^{-1} observed in IR is related to rocking vibrational mode (long alkyl chain, $-C_{12}H_{25}$) while 328, 1080 and 2888 cm^{-1} peaks in Raman indicate Pd-S vibration, C-C stretching and C-H stretching, respectively. Weight loss around 60% is associated to the loss of surfactant (dodecanethiol).

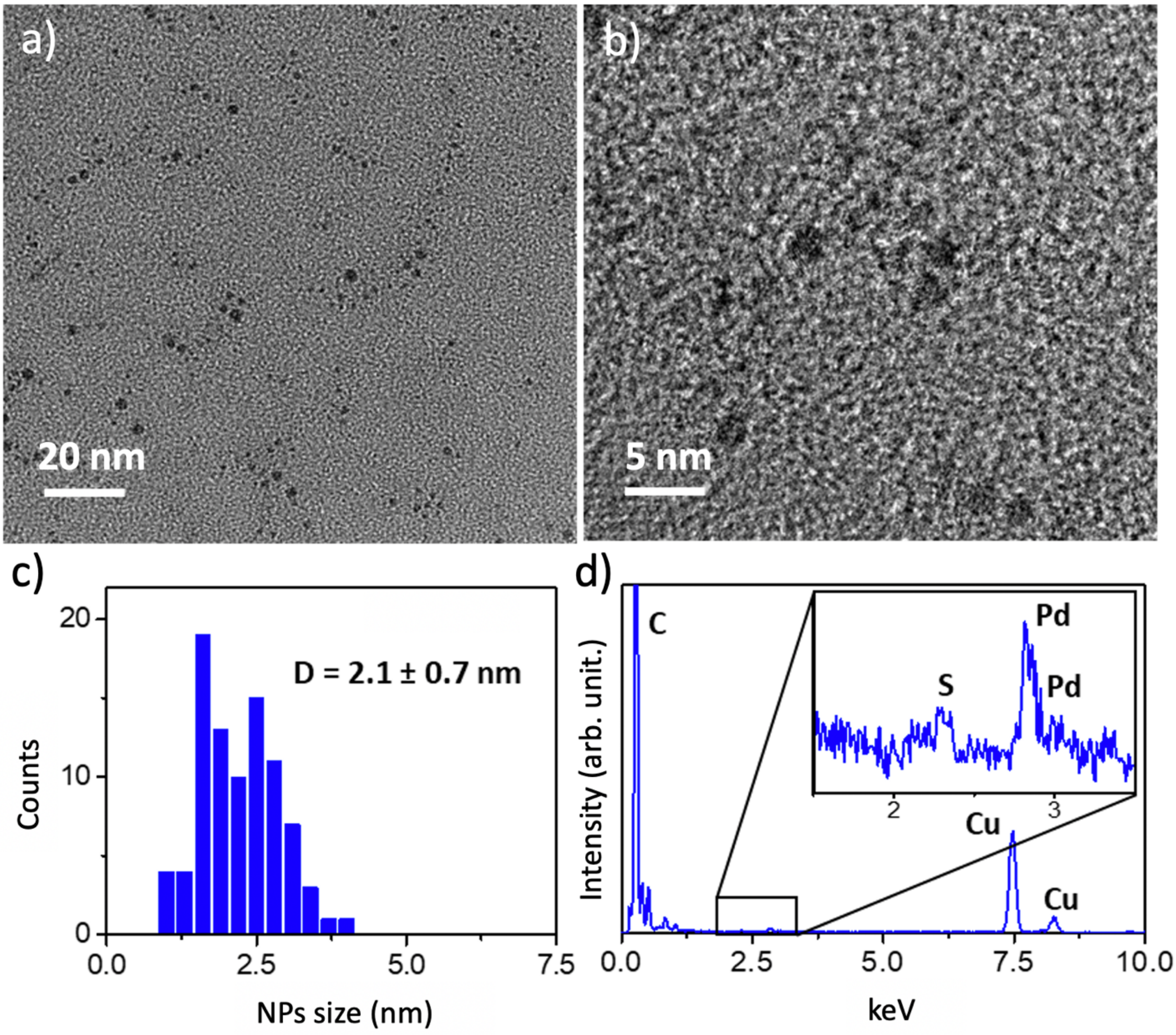

Supplementary Figure 2. (a-b) HRTEM images, **(c)** size distribution (D) of preformed Pd@SH-R nanoparticles measured by HRTEM (more than 80 NPs) with an average diameter (D) of 2.1 ± 0.7 nm, and **(d)** EDX of Pd@SH-R confirm the elemental composition of dodecanethiolate-stabilised PdNP. Scale bars are 20 and 5 nm, respectively.

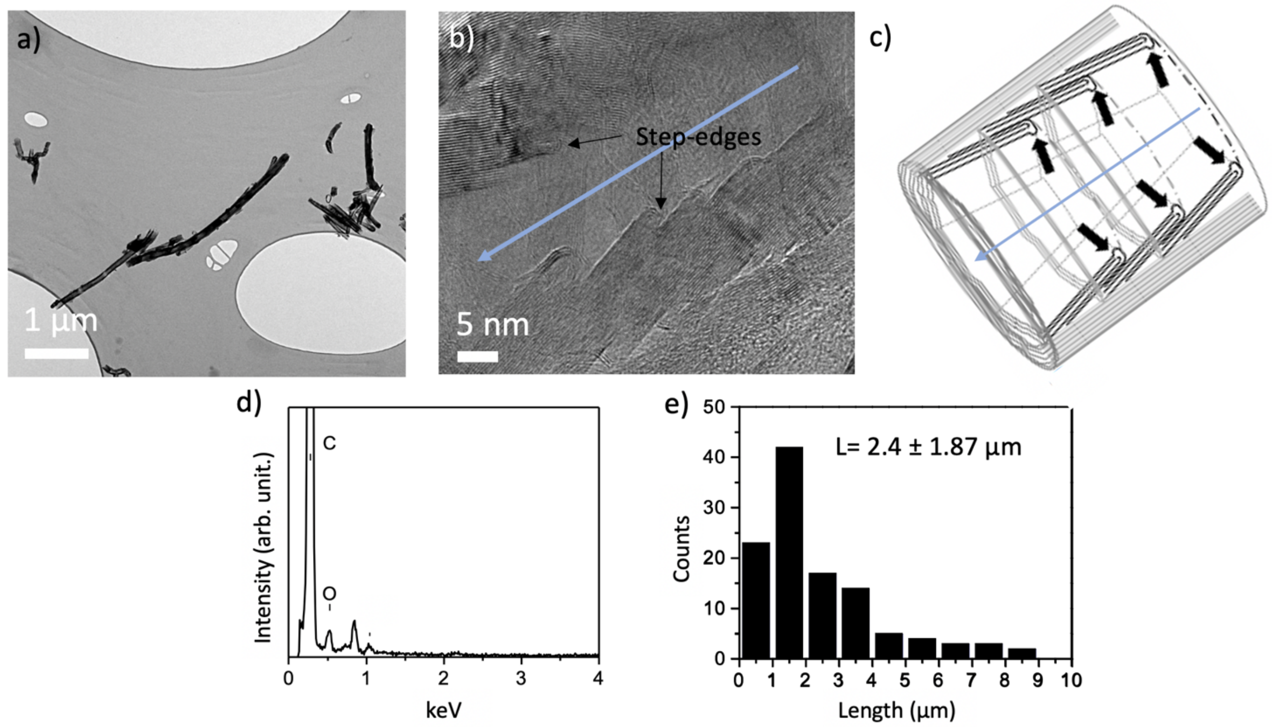

Supplementary Figure 3. (a-b) HRTEM images, **(c)** schematic illustration of CNF after milling showing with black arrows the step-edges, **(d)** EDX and **(e)** size distribution of milled CNF with an average length of 2.4 ± 1.9 µm measured by HRTEM.

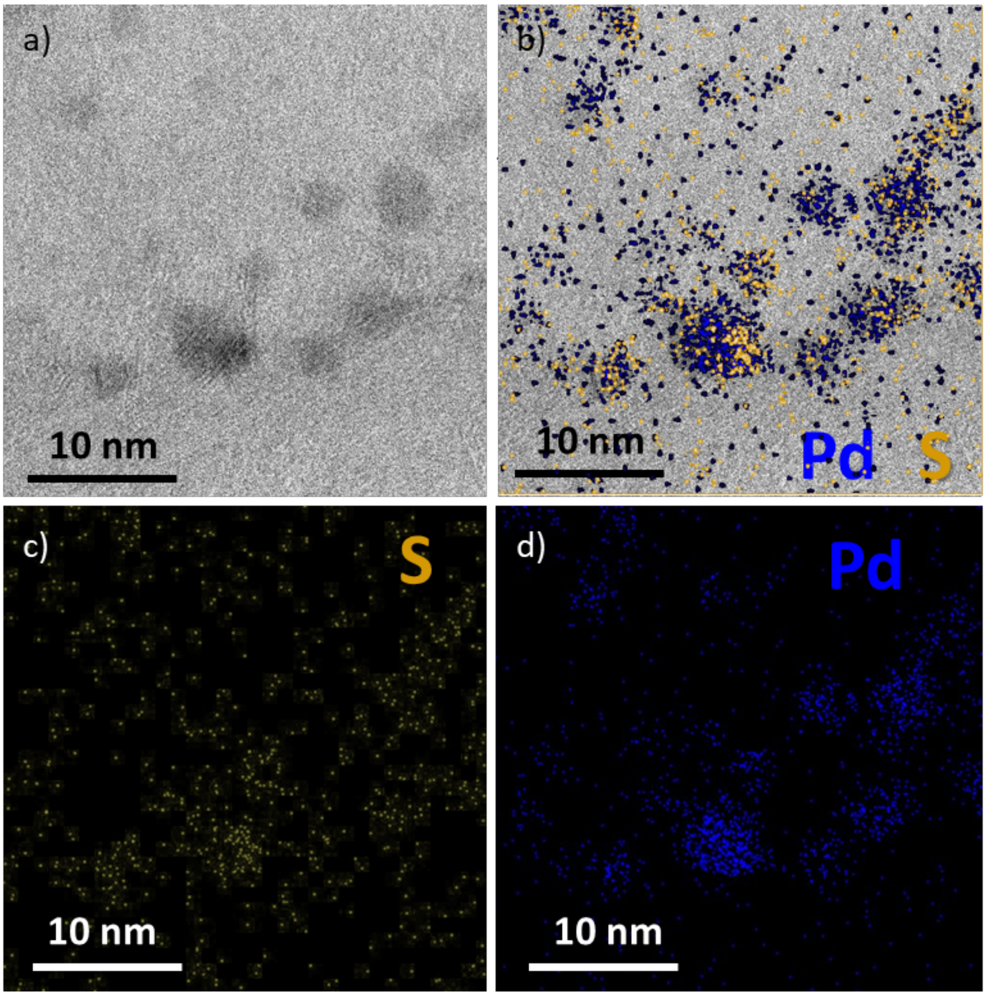

Supplementary Figure 4. (a) STEM and **(b-d)** EDS mapping images of PdS_x nanoparticles found inside of the internal cavities of CNF in the PdS_x/CNF material. Sulfur is shown in yellow and palladium in blue. All scale bars are 10 nm.

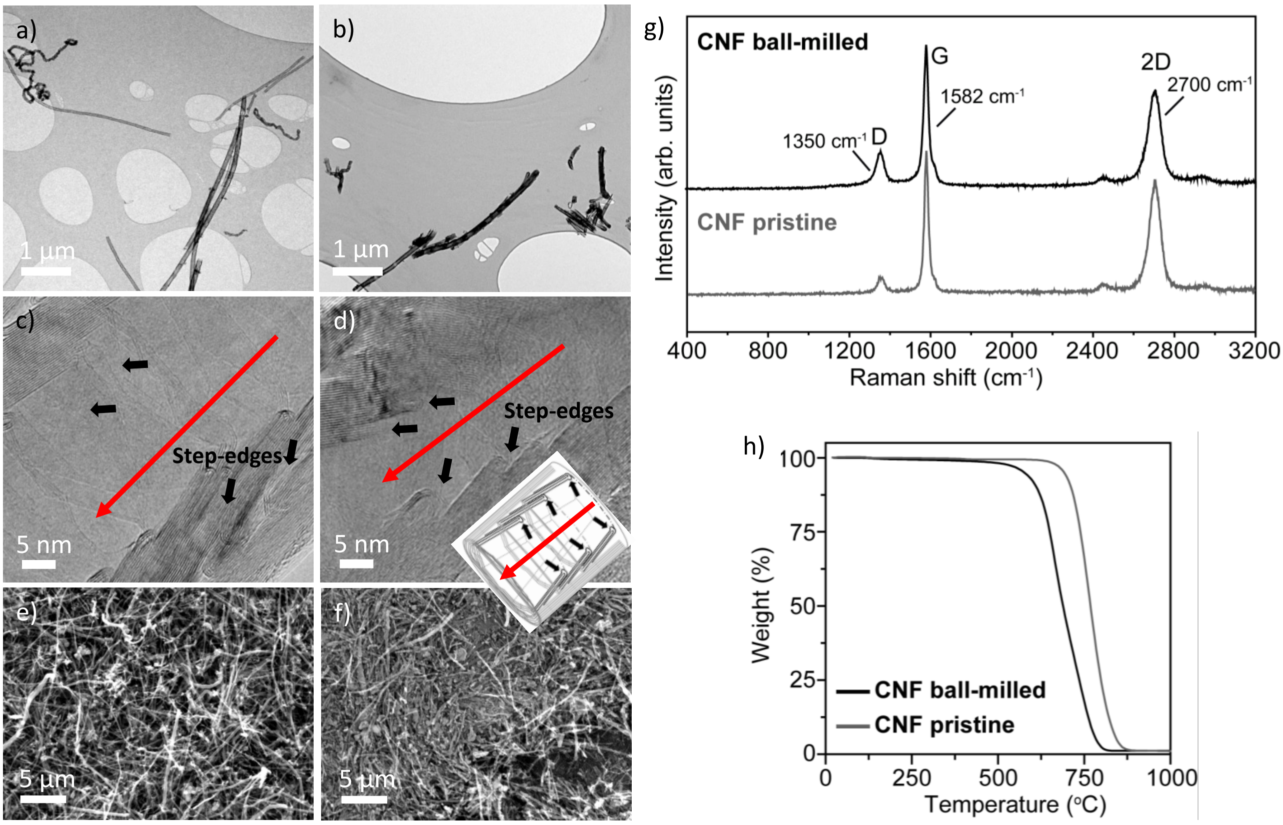

Supplementary Figure 5. HRTEM images of pristine CNF **(a,c)** and milled CNF **(b,d)**. Inset in d shows the schematic illustration of CNF indicating the presence of step-edges (black arrows) on the corrugated interior of CNF both before and after milling. FESEM images of **(e)** pristine CNF and **(f)** milled CNF, highlighting that nanofibers with shorten length are observed as result of the ball milling process **(g)** Raman spectrum of CNF ball-milled and pristine CNF showing a D, G and 2D band at 1350, 1550 and 2700 cm^{-1}. The ratio of defects is higher for CNF ball-milled giving an I_D/I_G = 0.25, whereas for pristine CNF is I_D/I_G = 0.12. **(h)** TGA measurements of ball-milled CNF and pristine CNF at a scan rate of 5°C/min in air showing thermal decomposition of CNF above 600°C whereas for CNF ball-milled the thermal decomposition starts earlier at 480°C, indicating the presence of defects in the structure.

a)

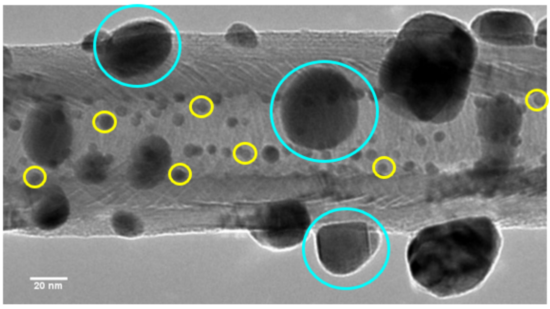

b)

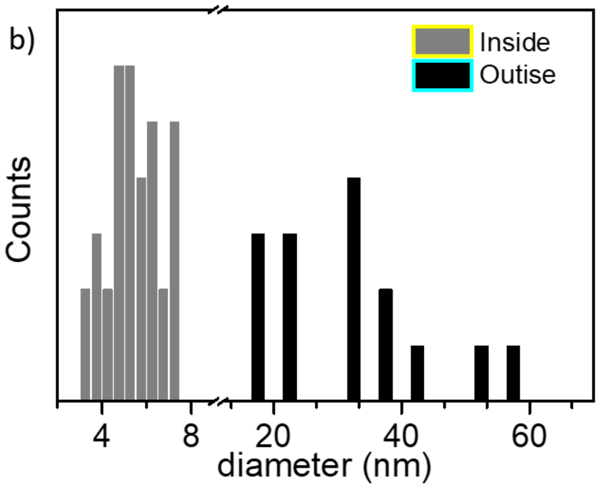

Supplementary Figure 6. (a) TEM image of PdS_x/CNF-pristine after thermal treatment at 300°C for 2 hours under vacuum. It can be observed that nanoparticles outside are larger than nanoparticles located inside when using pristine CNF which is in agreement with the nanoparticle size histogram **(b)**.

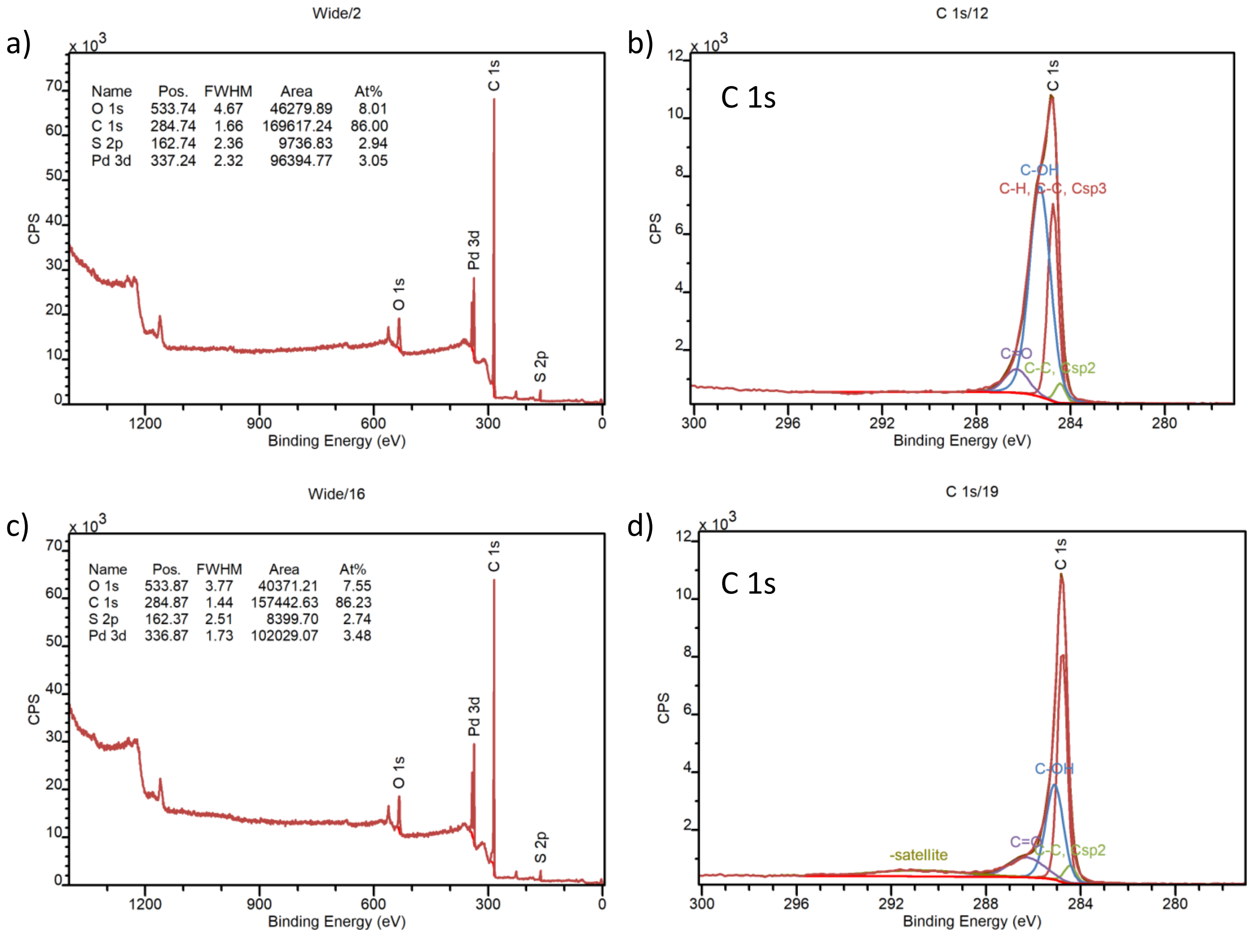

Supplementary Figure 7. XPS survey spectra and high-resolution spectra in the C1s region of **(a-b)** the material before heating (Pd@S-RH/CNF) and **(c-d)** the material after heating (PdS_x/CNF). Charge correction for the C 1s spectrum was set at the binding energy of 284.8 eV.

Supplementary Table 1. Some parameters on the interactions and compositions of C, S, and Pd in Pd@S-RH/CNF by the analysis of high resolution XPS spectra.

Name	Binding Energy (eV)	FWHM eV	Area (P) CPS.eV	Atomic composition, %
C1s C-C sp2	284.45	1.24	13894.8	2.81
C1s C-H, C_C sp3	284.75	0.52	3899.58	28.14
C1s C-O-C, C-O-H	285.4	1.05	8501.9	61.32
C1s C=O	286.31	1.18	1073.3	7.73
$S2p_{3/2}$ Metal-SH	162.87	0.96	407.47	49.06
$S2p_{1/2}$ Metal-SH	164.03	0.96	207.81	25.00
$S2p_{3/2}$ R-SH	163.49	0.88	142.79	17.18
$S2p_{1/2}$ R-SH	164.65	0.88	72.82	8.75
Pd $3d_{5/2}$ Pd(+)	336.53	1.24	19059.77	22.55
Pd $3d_{5/2}$ Pd(2+)	337.76	1.61	33167.77	39.24
Pd $3d_{3/2}$ Pd(+)	341.79	1.24	11785.97	13.94
Pd $3d_{3/2}$ Pd(2+)	343.02	1.61	20510.95	24.27

Supplementary Table 2. Some parameters on the interactions and compositions of C, S, and Pd in PdSx/CNF by the analysis of high resolution XPS spectra.

Name	Binding Energy (eV)	FWHM eV	Area (P) CPS.eV	Atomic composition, %
C1s C-C sp2	284.35	0.60	422.473	4.10
C1s C-H, C_C sp3	284.78	0.47	4224.73	40.96
C1s C-O-C, C-O-H	285.10	0.87	3212.28	31.14
C1s C=O	286.31	1.76	1365.78	13.23
C1s satellite	290.84	4.70	1096.39	10.58
$S2p_{3/2}$ Metal-Sulfide	162.01	1.61	1327.74	29.28
$S2p_{1/2}$ Metal-Sulfide	163.17	1.61	795.86	14.92
$S2p_{3/2}$ Metal-SH	162.54	1.27	2655.69	36.97
$S2p_{1/2}$ Metal-SH	163.70	1.27	1352.96	18.84
Pd $3d_{5/2}$ Pd(2+)	336.99	2.03	41828.92	61.93
Pd $3d_{3/2}$ Pd(2+)	342.25	2.03	25716.95	38.07

Supplementary Table 3. Atomic composition of S and Pd in obtained from the corresponding high resolution XPS of S 2p and Pd 3d.

**Atomic composition % (At%)**	**Before heating**	**After heating**
S $2p^3$ metal sulfide	-	44.20
S $2p^3$ thiol bound to metal	74.06	55.81
S $2p^3$ thiol R-SH	25.93	-
Pd $3d^5$ Pd^+	36.49	-
Pd $3d^5$ Pd^{2+}	63.51	100

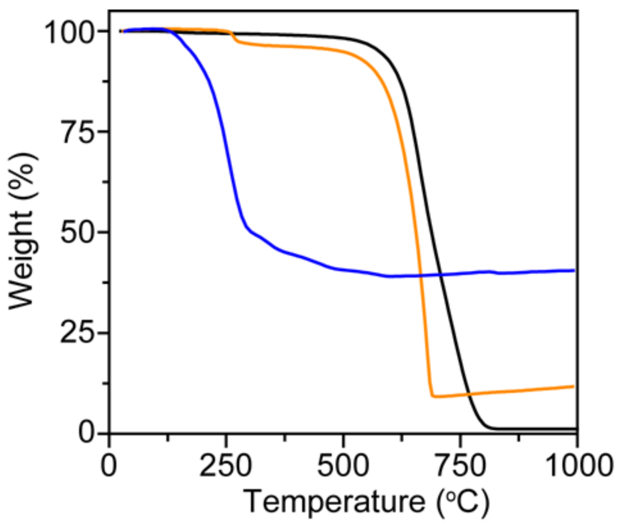

Supplementary Figure 8. Thermal gravimetric analysis in air of preformed palladium nanoparticles (Pd@SH-R , blue), CNF (black) and PdS_x/CNF material (orange).

Supplementary Table 4. Electrochemical parameters for HER catalysts PdS_x/CNF, Pd/AC and CNF.

**Sample**	**Activation scans (between -0.9V and 1.2V)**	**η (mV)@10 mA/cm^2**	**η variation (mV) @ 10mA/cm^2**	**Tafel Slope (mV/dec)**	**E_{onset} (mV)**
PdS_x/CNF	0	296.3	**–**	41	-150
	200	22.0	**274.3**	34	0
	500	26.0	**4**	34	0
	1000	25.0	**1**	34	0
Pd/AC (20 wt%)	0	40.5	**–**	50	0
	200	21.8	**18.7**	20	0
CNF	0	812.4	**–**	214	- 550
	200	552.6	**259.8**	182	- 300

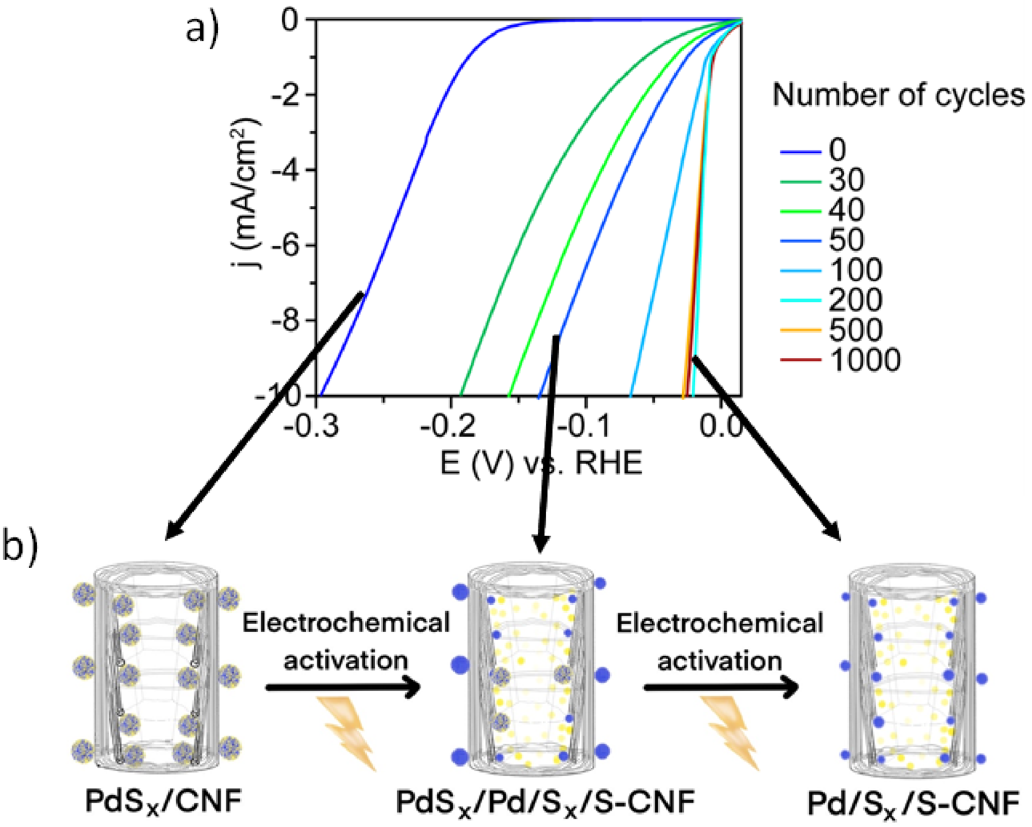

Supplementary Figure 9. (a) HER-LSV measurements of PdS_x/CNF during the activation experiment after several cycles. **(b)** Representation of the PdS_x/CNF transformations along with the number of cycles.

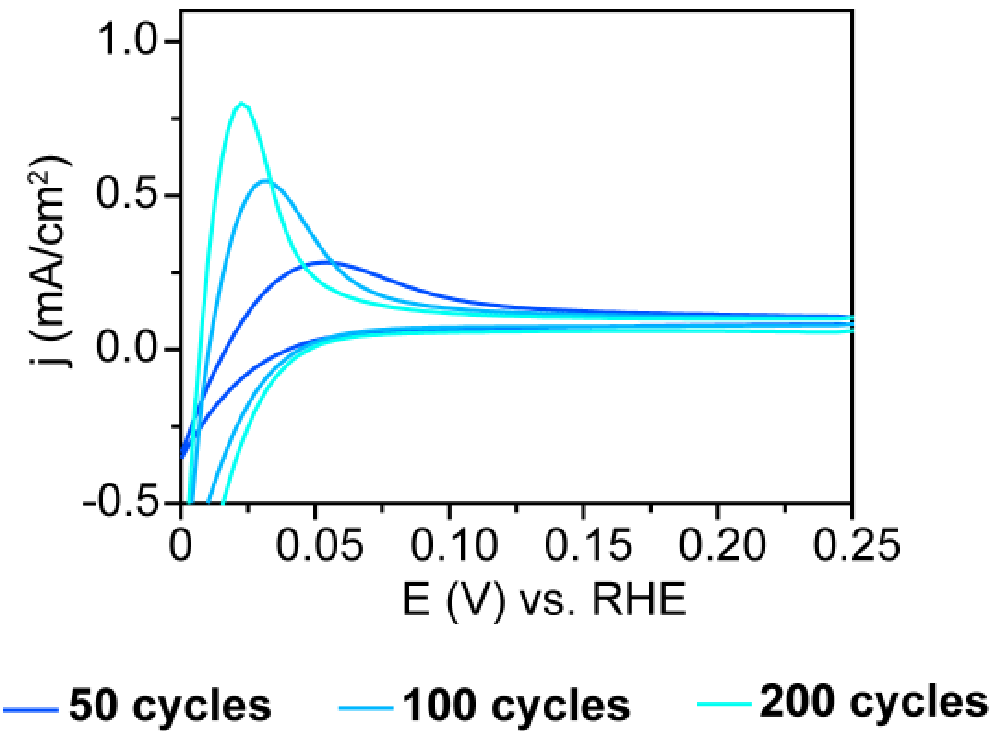

Supplementary Figure 10. Magnification of the CV curves of PdS_x/CNF during the activation experiment showing the increase of H desorption peak at different cycling (50, 100 and 200 cycles). Cyclic voltammetry (CV) measurements were recorded at a scan rate of 10 mV/s in 0.1 M $HClO_4$ under N_2 atmosphere.

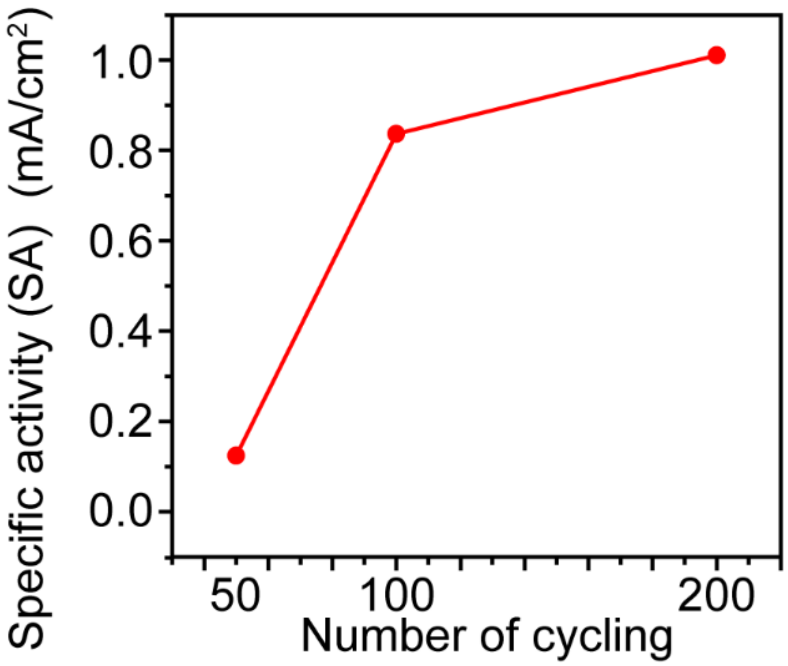

Supplementary Figure 11. Specific activity (mA/cm^2) calculated for the PdS_x/CNF material at different number of cycles (50, 100 and 200 cycles).

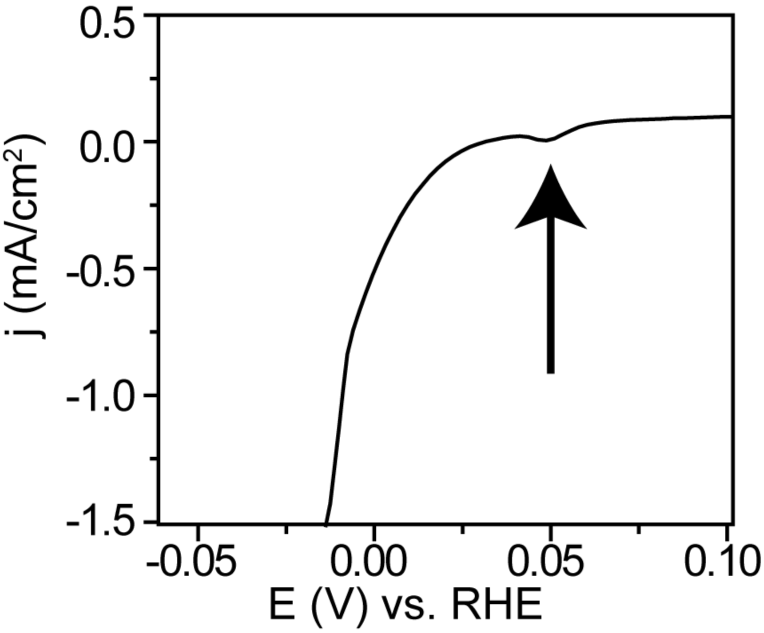

Supplementary Figure 12. Zoom of the LSV curve of the Pd/S_x/S-CNF showing the appearance of the H_{UPD} peak after 200 potential cycles.

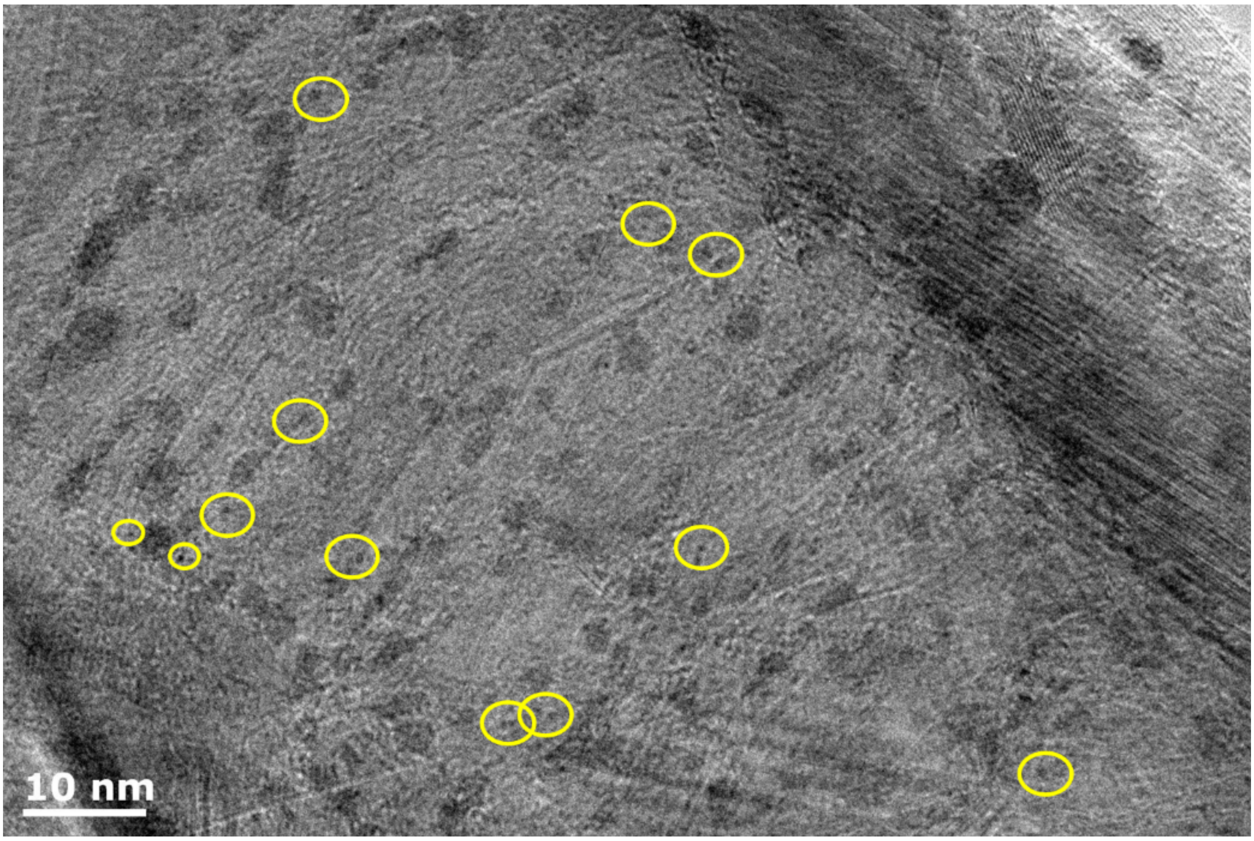

Supplementary Figure 13. High magnification TEM image of the interior of CNF in Pd/S_x/S-CNF (after 200 HER cycles) where nanoparticles of less than 1 nm size can be observed (yellow circles). Scale bar is 10 nm.

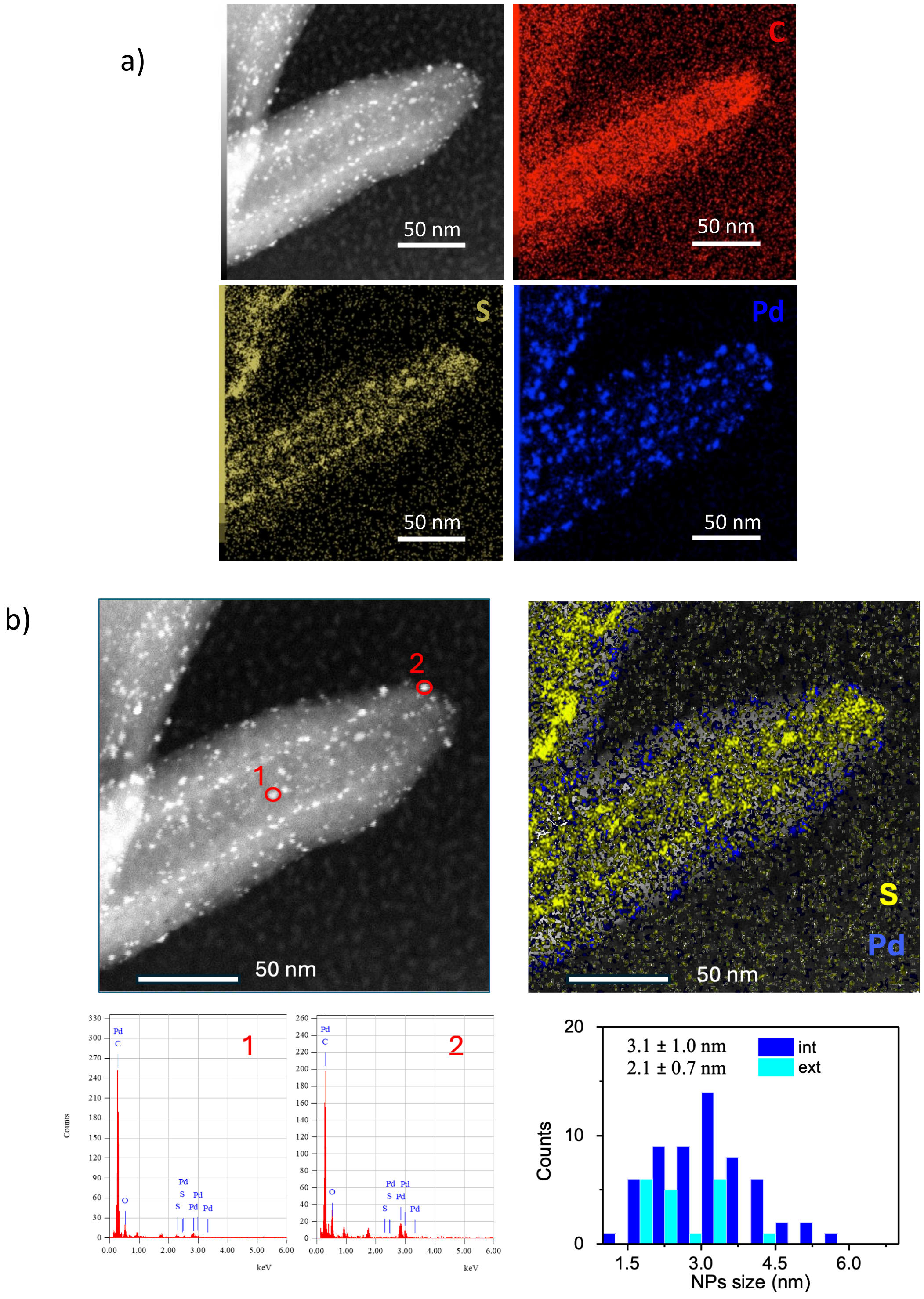

Supplementary Figure 14. Electrochemically activated material (Pd/S_x/S-CNF) after 1000 potential cycles: (a) Dark-field TEM and EDS elemental mapping images showing the distribution of carbon (red), sulfur (yellow), and palladium (blue); and (b) overlaid sulfur (yellow) and palladium (blue) EDS elemental mappings, EDX analysis, and particle size distribution for confined and unconfined nanoparticles after 1000 potential cycles.

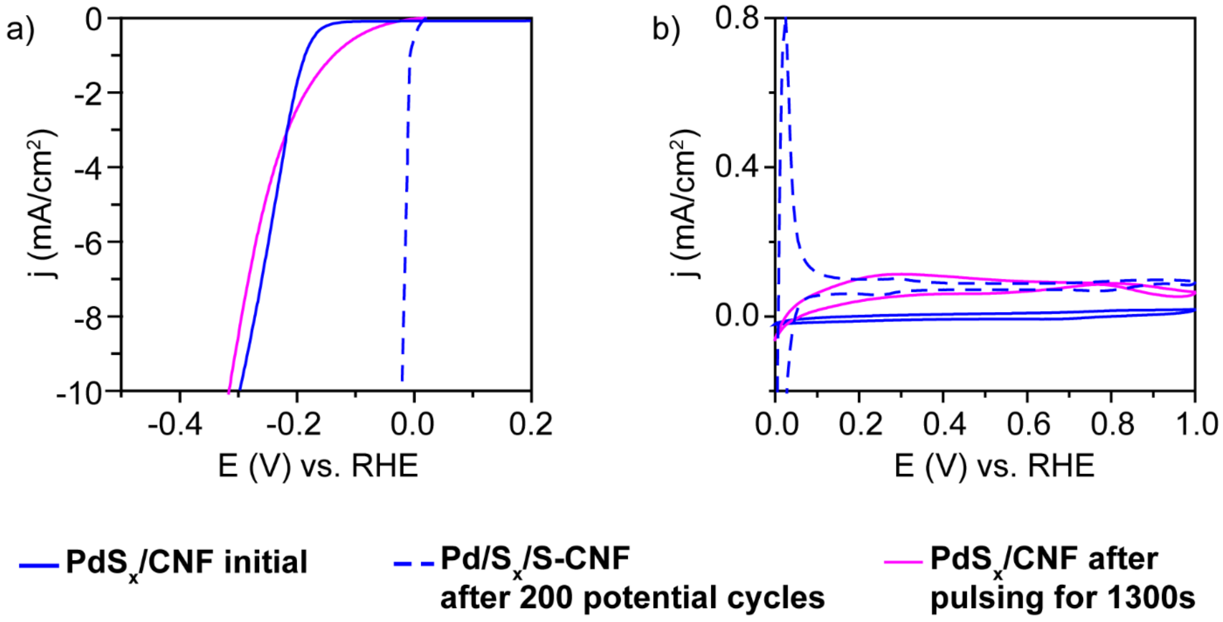

Supplementary Figure 15. (a) HER-LSV polarization curves and **(b)** CV at scan rate of 50 mV/s of PdS_x/CNF as initial, activated after 200 potential cycles (Pd/S_x/S-CNF) and after potential step experiment by alternating the potential for 1s from -0.9V to 1.2V, respectively, for a total duration of 1300 s.

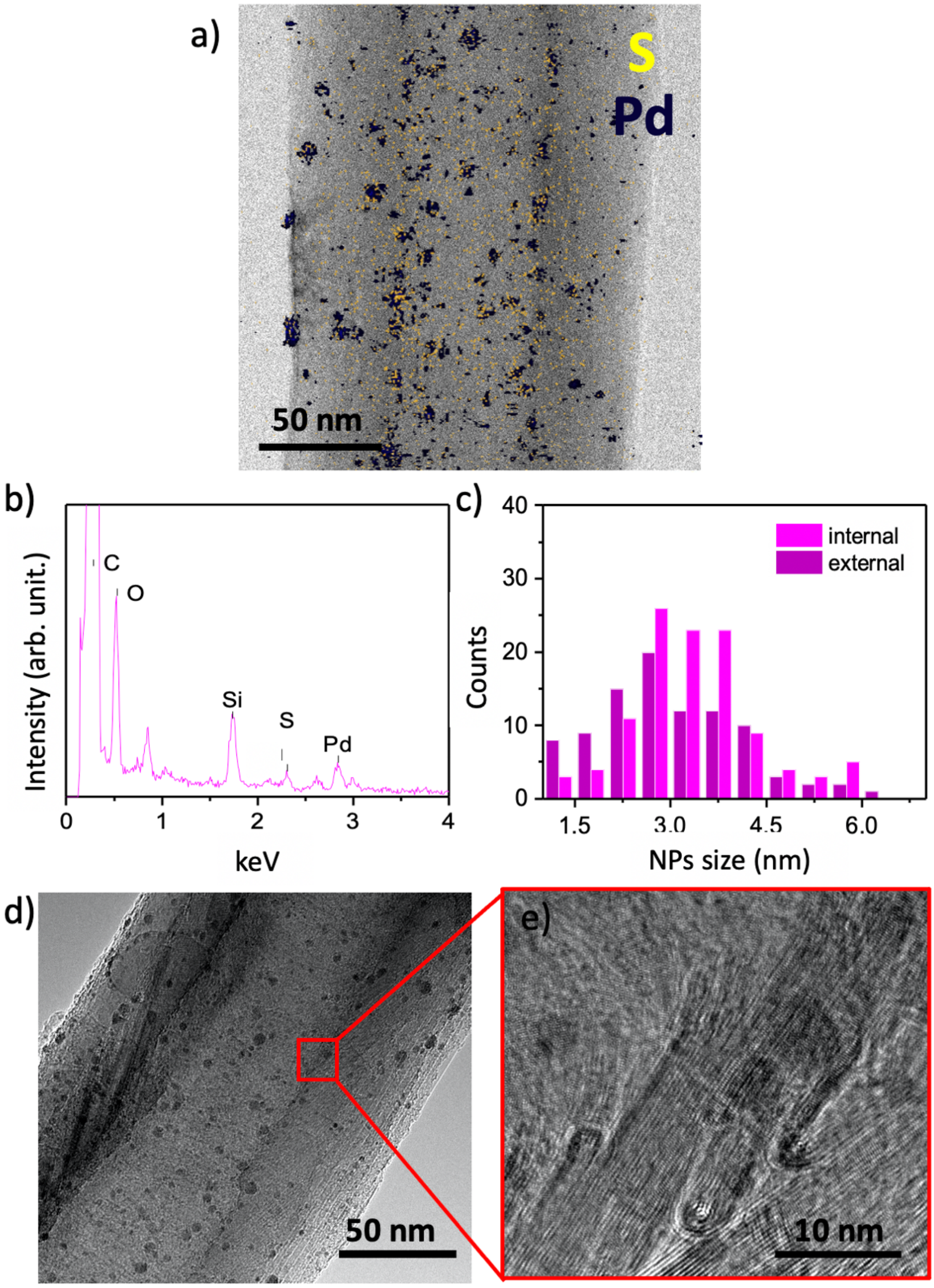

Supplementary Figure 16. **(a)** STEM-EDS mapping image, being Pd (blue) and S (yellow), **(b)** EDS spectra and **(c)** particle size distribution of PdS_x/CNF after a potential step experiment by alternating the potential from -0.9V (1 s) to 1.2V (1 s) for a total duration of 1300s. Particle size distribution shows 3.07 ± 0.81 nm external diameter and 2.70 ± 0.78 nm of internal diameter. **(d)** An HRTEM representative image of PdS_x/CNF after potential step experiments and **(e)** a high magnification HRTEM image of the rectangle area in (d) showing that the internal graphitic structure is still well intact.

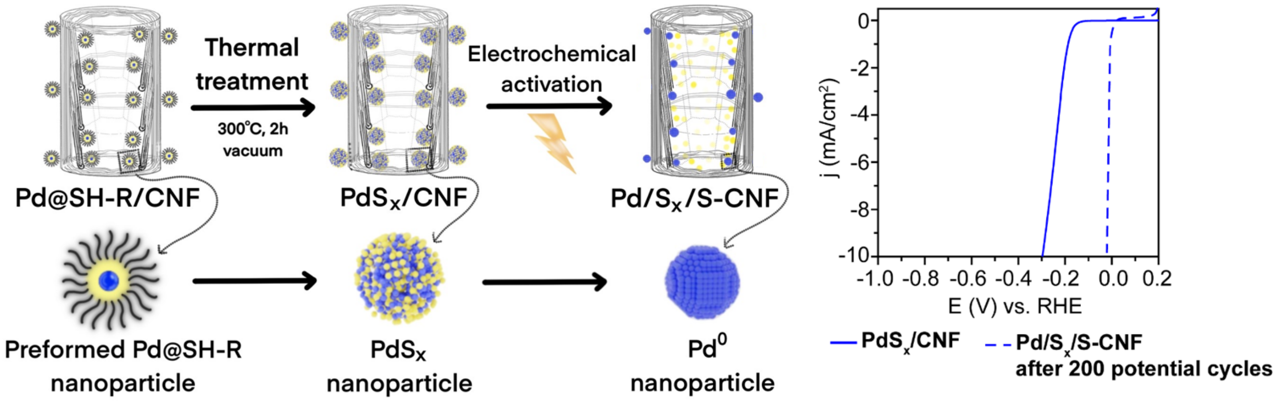

Supplementary Figure 17. Schematic illustration of the thermal treatment and electrochemical activation process for the material under investigation. The initial transformation in the nature of Pd@SH-R/CNF can be attributed to its exposure to high temperature, which converts preformed Pd@SH-R nanoparticles into amorphous PdS_x nanoparticles. Subsequently, upon subjecting PdS_x/CNF material to an electrochemical potential window ranging from -0.9 V to 1.2 V, further transformations occur resulting in the release of sulfur atoms during the formation of crystalline Pd^0 nanoparticle from amorphous PdS_x nanoparticles, giving rise to the Pd/S_x/S-CNF material. The latter leads to an improvement in the HER activity after 200 cycles (i. e. electrochemical activation), as seen in the graph at the right.

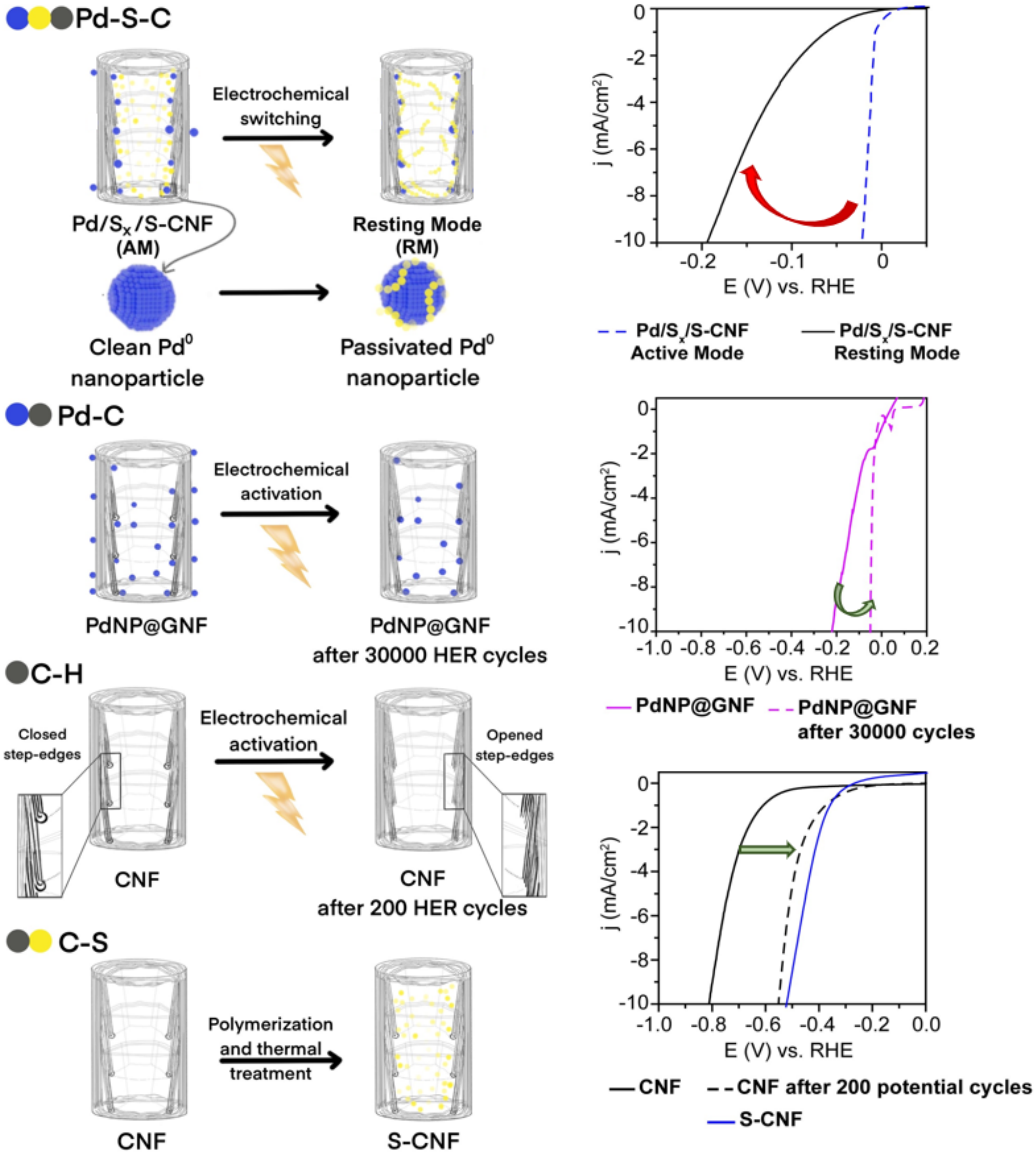

Supplementary Figure 18. Schematic illustration of the electrochemical switching ability for the material under investigation (Pd/S_x/S-CNF) and its controls PdNP@GNF, CNF and S-CNF. It is illustrated how Pd/S_x/S-CNF in active mode undergoes a switching ON/OFF HER activity if it is subjected to oxidation (i. e. several potential cycles from -0.2V to 0.4V). The control material, PdNP@GNF, which consists of Pd(0) nanoparticles supported on ball-milled GNF (being GNF same as our CNF) did not exhibited a regression in the activity after the same potential cycling protocol (i. e. from -0.2V to 0.4V) due to the absence of sulfur.

On the contrary, it incremented its activity which was maintained even after 30000 HER cycles.[2] The same was observed for the control CNF, which also incremented its activity after the same potential cycling protocol. This observation agrees with the opening of the step-edges.[2] The control material containing only sulfur and carbon (S-CNF) was also synthesized. In the S-CNF material, sulfur is covalently bonded to CNF. The overpotential of S-CNF is similar to that of CNF after 200 cycles. The electrochemical HER behavior of each material can be seen in the LSV curves at the right.

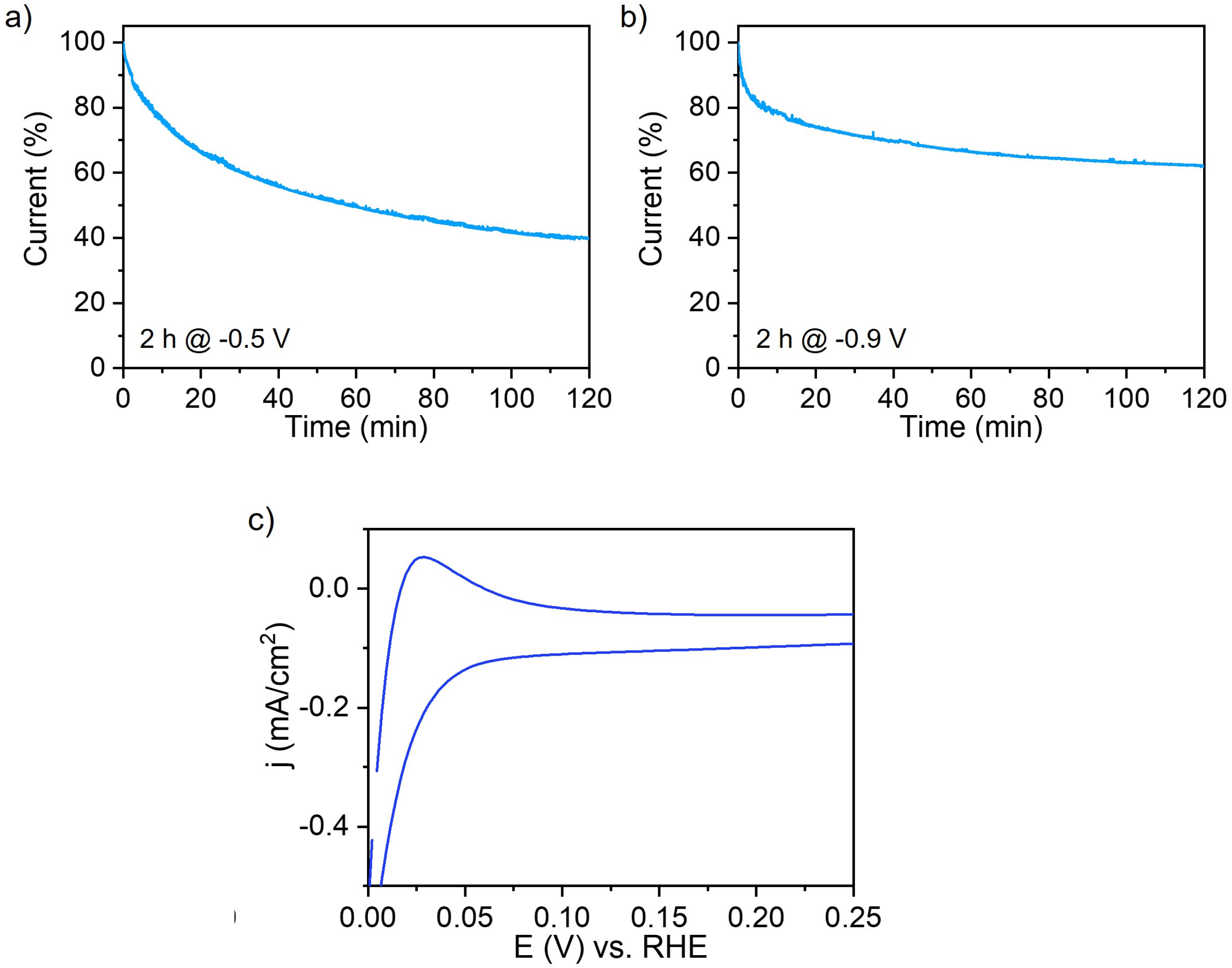

Supplementary Figure 19. Chronoamperometry measurements at fixed potentials of a) -0.5 and b) -0.9V and c) cyclic voltammogram after chronoamperometry experiment showing the H desorption peak of Pd/S_x/S-CNF.

Supplementary Table 5. Electrochemical HER parameters for other electrocatalyst materials reported in literature.

**Catalyst**	**Electrolyte**	**Overpotential (mV) @ 10 mAcm^{-2}**	**Tafel slope (mV dec^{-1})**	**Ref**
Pd/S_x/S-CNF	0.1 M HClO4	22	34	This work
PdNP@GNF after 30K cycles	0.1 M HClO4	50	52	[2]
MoN-NC NPs	0.5 M H_2SO_4	62	54	[3]
n-Pd@NDCDs	0.5 M H_2SO_4	291	135	[4]
Pd@PANI/Au	0.5 M H_2SO_4	60	35	[5]
Mn_3N_2/PdO	0.5 M H_2SO_4	45	50	[6]
Pd/HOPG	0.1 M H_2SO_4	150	118	[7]
Pd-MoS_2/MWCNT after 500 cycles	0.5 M H_2SO_4	125	54	[8]

All potentials are reported vs RHE.

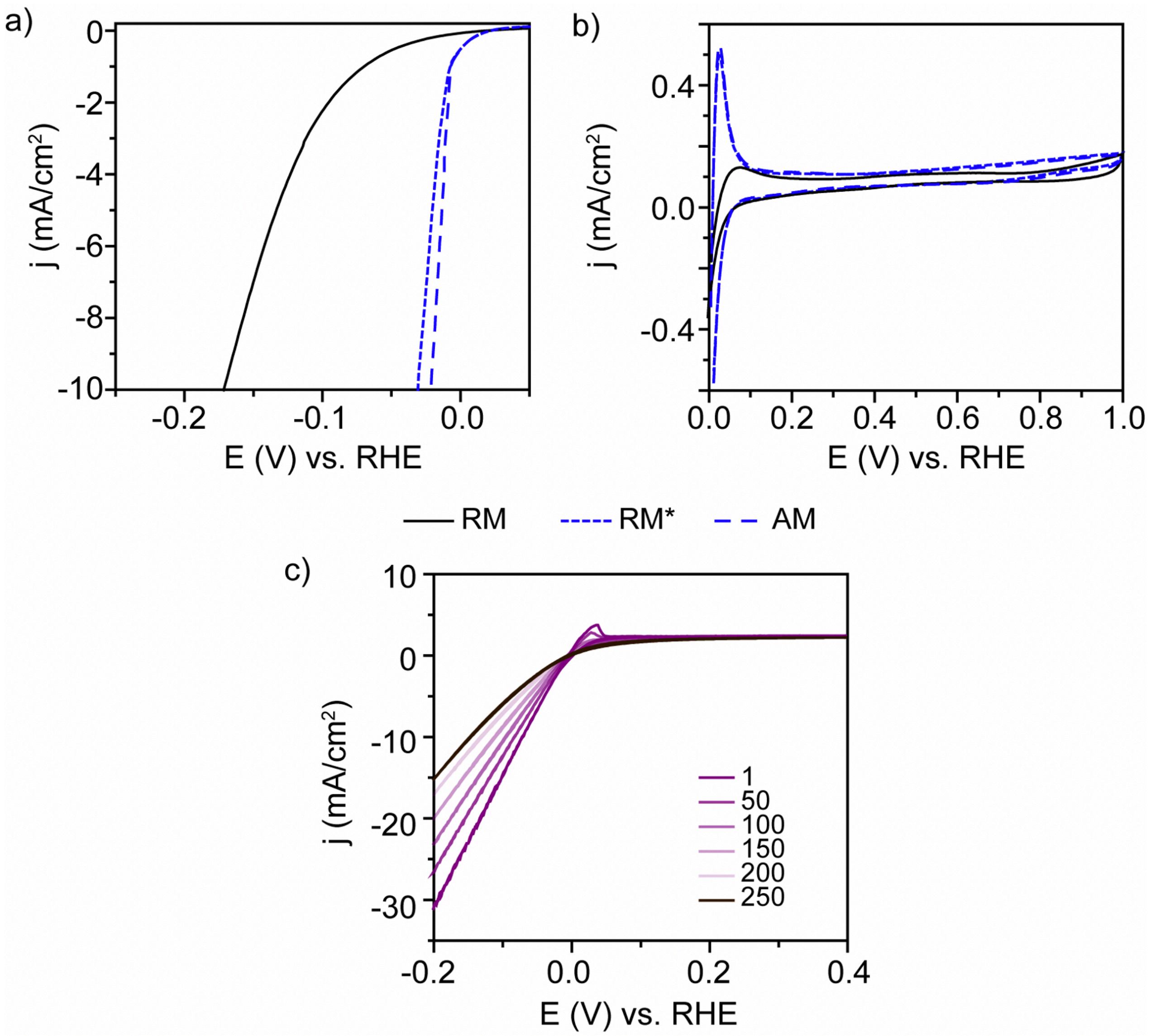

Supplementary Figure 20. (a) HER-LSV at 50 mV/s, and **(b)** CVs of $Pd/S_x/S$-CNF as in AM (blue dash line), $Pd/S_x/S$-CNF as in RM cycled from 0.4V to -0.2V for 250 cycles (black solid line), and $Pd/S_x/S$-CNF as in RM* after 1 wide potential cycling to activate material again (blue small, dashed line), **(c)** CV from 0.4 to -0.2V of $Pd/S_x/S$-CNF from scan 1 (purple) to scan 250 (dark purple) show the induction of the resting mode (RM) from the active mode (AM).

Supplementary Table 6. Electrochemical HER parameters for the different modes of $Pd/S_x/S$-CNF after OCP.

	E_{onset} (mV)	η (mV) @ 10mA/cm^2	Tafel Slope (mV/dec)
$Pd/S_x/S$-CNF (AM)	0	22	34
RM	20	193	113
RM*	0	40	56

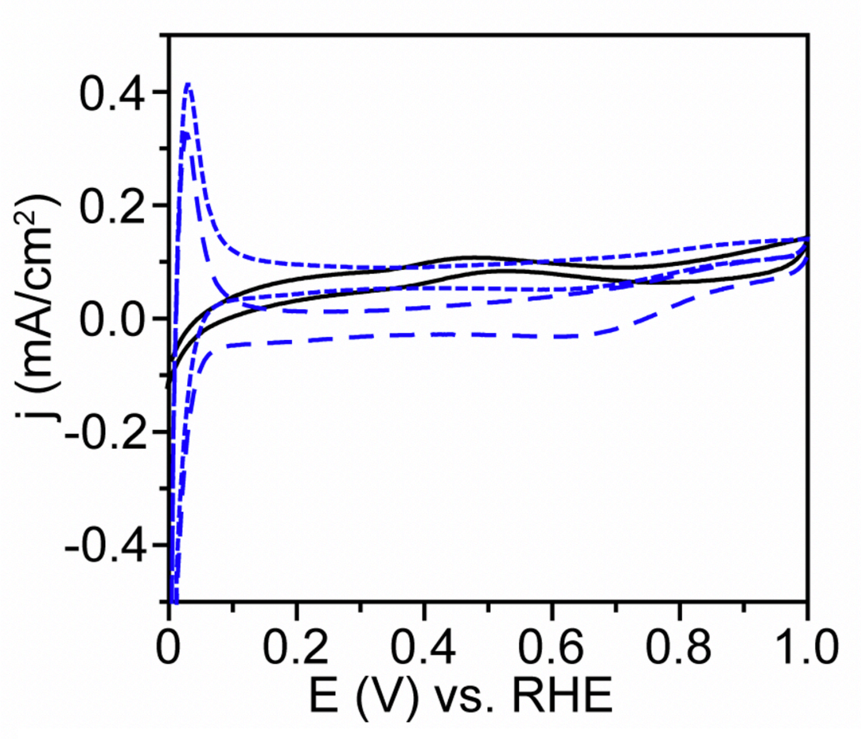

Supplementary Figure 21. CV of Pd/S_x/S-CNF (AM) as blue dash line, Pd/S_x/S-CNF as in the resting mode after OCP (RM) (black solid line) and Pd/S_x/S-CNF reactivated from the resting mode (RM*) (blue small, dashed line).

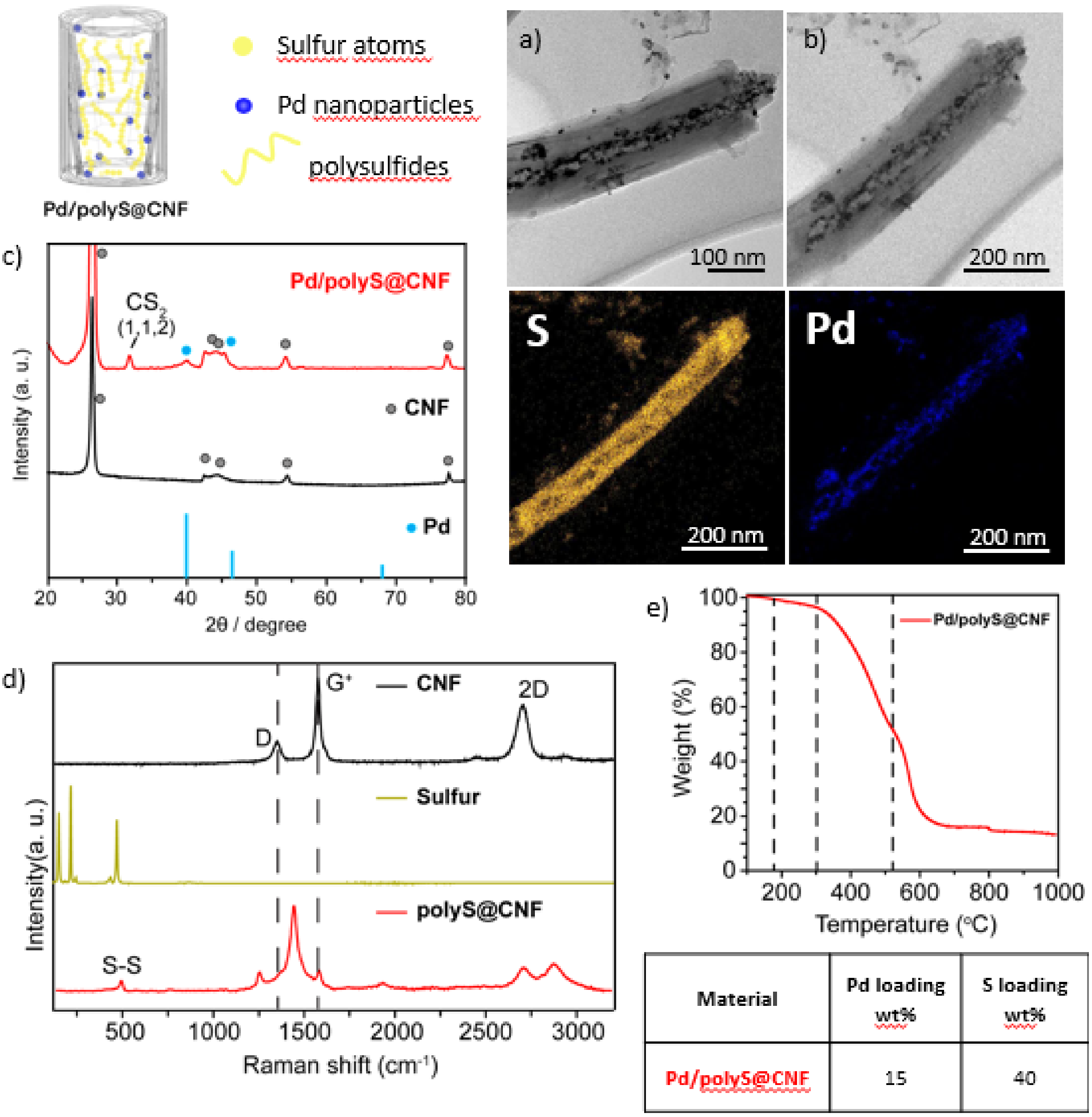

Material	Pd loading wt%	S loading wt%
Pd/polyS@CNF	15	40

Supplementary Figure 22. (a,b) HRTEM images and STEM-EDX mapping of Pd/polyS@CNF. The yellow color in the STEM-EDX image reveals the presence of sulfur and blue color for palladium. At the left there is a schematic representation of the material. In the Pd/polyS@CNF composite, sulfur and palladium are predominantly confined within the internal channel of CNF. This leads to poorly defined palladium nanoparticles as they tend to aggregate with sulfur. **(c)** XRD measurements of the Pd/polyS@CNF material showing CNF as control and the pattern of Pd.[9] Carbon disulfide reference code: 96-150-6831. XRD measurements confirmed the presence of crystalline palladium metal as a peak was shown at 39.5° corresponding to the (111) Pd. **(d)** Raman measurements of polyS@CNF material showing the main characteristic peaks. CNF and sulfur powder are also measured as controls. Raman spectrum of CNF showed a typical pattern of graphitized carbon, in which three characteristic bands are observed; (i) the G^+ band at 1580 cm^{-1} corresponding a bond stretching vibration of a pair of sp^2 sites called graphitic cluster lattice

vibration mode with E2g symmetry, (ii) the D band at 1350 cm^{-1} corresponding an A1g breathing vibration of a 6-fold aromatic ring that is activated by disordered carbon, and (iii) the 2D band around 2700 cm^{-1} corresponding to the second order of zone-boundary phonons.[10] Raman analysis of the polyS@CNF gave a main peak assigned to the carbon-carbon unsaturated bond (C = C bond) at 1444 cm^{-1}, and another peak at 505 cm^{-1} assigned to the disulfide linkage in the carbon-sulfur polymer.[1] Note that crystalline sulfur contributes several peaks in the region from 100 to 500 cm^{-1}, which are not seen in the sample, but only the disulfide linkage peak remains.[11] **(e)** TGA measurement and quantification of the components by weight, according to TGA, of Pd/polyS@CNF. The sample shows a significant weight loss at around 300 ℃ corresponding to the sulfur-carbon species and a residual weight form 800°C related to the amount of palladium metal.

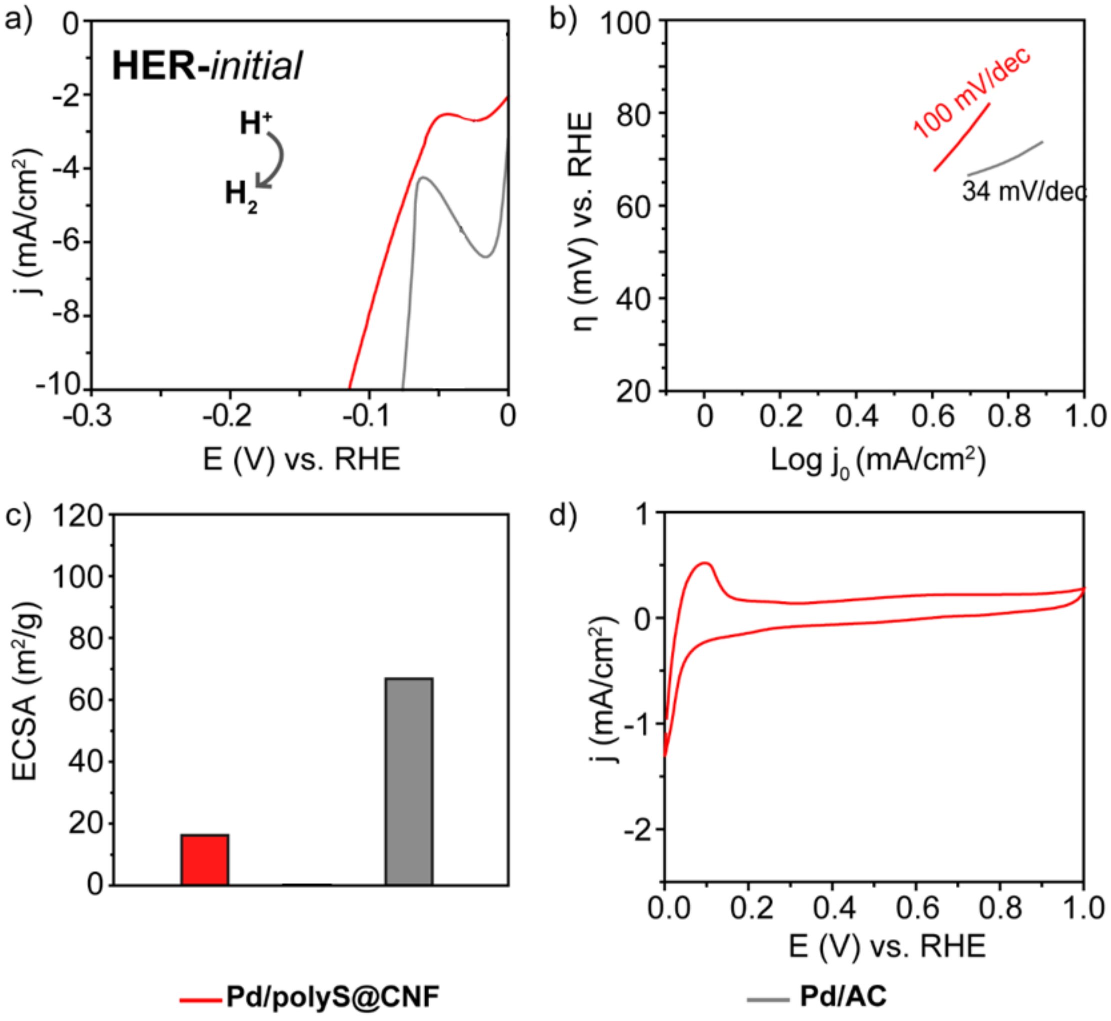

Supplementary Figure 23. **(a)** The linear polarization curves and **(b)** Tafel plots were obtained for the Pd/polyS@CNF catalyst. **(c)** A comparison of the ECSA parameter of as-prepared Pd/polyS@CNF and Pd/AC benchmark catalyst. **(d)** CV measurements for the Pd/polyS@CNF catalyst in N_2-saturated 0.1 M $HClO_4$ aqueous solution. Scan rate of 50 mV s^{-1}.

Supplementary Table 7. Electrochemical HER parameters for Pd/polyS@CNF catalyst compared with the activity of the resting mode (RM) and the benchmark catalysts measured under the same conditions.

Catalyst	E_{onset} (mV)	Overpotential (mV) at -10 mA/cm^2	Tafel slope (mV/dec)
Pd/polyS@CNF	-50	115	100
RM	-20	193	113
Pd/AC	-62	76	34

All potentials are reported vs RHE.

Supplementary Table 8. ECSA, MA and SA values for the active mode (AM) and reactivated mode (RM*) of Pd/S_x/S-CNF compared with the benchmark Pd/AC (20 wt%).

	ECSA (m^2/g)	MA (A/mg)	SA (mA/cm^2)
Pd/S_x/S-CNF (AM)	95.8	0.96	1.01
RM*	74.8	0.41	0.55
Pd/AC (20 wt%)	66	0.12	0.18

References SI

[1] High sulfur-containing carbon polysulfide polymer as a novel cathode material for lithium-sulfur battery, Y. Zhang, Y. Peng, Y. Wang, J. Li, H. Li, J. Zeng, J. Wang, B. J. Hwang, J. Zhao, *Scientific Reports* **2017**, *7*, 11386.

[2] Palladium nanoparticles hardwired in carbon nanoreactors enable continually increasing electrocatalytic activity during the hydrogen evolution reaction, M. Aygün, M. Guillen-Soler, J. M. Vila-Fungueiriño, A. Kurtoglu, T. W. Chamberlain, A. N. Khlobystov, M. del Carmen Gimenez-Lopez, *ChemSusChem* **2021**, *14*, 4973-4984.

[3] Electrocatalytic performance of carbon dots/palladium nanoparticles composite towards hydrogen evolution reaction in acid medium, P. Chandrasekaran, T. N. Jebakumar Immanuel Edison, M. G. Sethuraman, *International Journal of Hydrogen Energy* **2020**, *45*, 28800-28811.

[4] Electrocatalytic activity of MWCNT supported Pd nanoparticles and MoS_2 nanoflowers for hydrogen evolution from acidic media, E. Heydari-Bafrooei, S. J. Askari, *International Journal of Hydrogen Energy* **2017**, *42*, 2961-2969.

[5] Palladium nanoparticles supported on highly oriented pyrolytic graphite: preparation, reactivity and stability, W. Ju, T. Brülle, M. Favaro, L. Perini, C. Durante, O. Schneider, U. Stimming, *ChemElectroChem* **2015**, *2*, 547-558.

[6] Palladium-coated polyaniline nanofiber electrode as an efficient electrocatalyst for hydrogen evolution reaction, B. Kurt Urhan, H. Öztürk Doğan, T. Öznülüer Özer, Ü. Demir, *International Journal of Hydrogen Energy* **2022**, *47*, 4631-4640.

[7] Palladium oxide decorated transition metal nitride as efficient electrocatalyst for hydrogen evolution reaction, K. C. Majhi, M. Yadav, *Journal of Alloys and Compounds* **2021**, *855*, 157511.

[8] Enhancing electrocatalytic activity for hydrogen evolution by strongly coupled molybdenum nitride@ nitrogen-doped carbon porous nano-octahedrons, Y. Zhu, G. Chen, X. Xu, G. Yang, M. Liu, Z. Shao, *ACS Catalysis* **2017**, *7*, 3540-3547.

[9] Green preparation and catalytic application of Pd nanoparticles, L. Xu, X.-C. Wu, J.-J. Zhu, *Nanotechnology* **2008**, *19*, 305603.

[10] Characterizing Graphene, Graphite, and Carbon Nanotubes by Raman Spectroscopy, M. S. Dresselhaus, A. Jorio, R. Saito, *Annual Review of Condensed Matter Physics* **2010**, *1*, 89-108.

[11] Porous spherical polyacrylonitrile-carbon nanocomposite with high loading of sulfur for lithium–sulfur batteries, H. Sohn, M. L. Gordin, M. Regula, D. H. Kim, Y. S. Jung, J. Song, D. Wang, *Journal of Power Sources* **2016**, *302*, 70-78.
